# Charting the brain networks of impulsivity: Meta-analytic synthesis, functional connectivity modelling, and neurotransmitter associations

**DOI:** 10.1162/imag_a_00295

**Published:** 2024-09-25

**Authors:** Martin Gell, Robert Langner, Vincent Küppers, Edna C. Cieslik, Theodore D. Satterthwaite, Simon B. Eickhoff, Veronika I. Müller

**Affiliations:** Department of Psychiatry, Psychotherapy and Psychosomatics, Medical Faculty, RWTH Aachen University, Aachen, Germany; Institute of Neuroscience and Medicine (INM-7: Brain & Behaviour), Research Centre Jülich, Jülich, Germany; Institute of Systems Neuroscience, Medical Faculty and University Hospital Düsseldorf, Heinrich Heine University Düsseldorf, Düsseldorf, Germany; Department of Nuclear Medicine, University Hospital and Medical Faculty, University of Cologne, Cologne, Germany; Department of Psychiatry, Perelman School of Medicine, University of Pennsylvania, Philadelphia, PA, United States

**Keywords:** impulsivity, meta-analysis, delay discounting, response inhibition, functional connectivity, neurotransmitters

## Abstract

Impulsivity is a multi-dimensional construct that plays a crucial role in human behaviour and is believed to be a transdiagnostic marker of several psychiatric disorders. However, given its multi-faceted nature, investigations of its neural correlates are challenging and systematic comparisons across dimensions are lacking. In this study, we used a comprehensive multi-modal approach to investigate the functional network organisation of two dimensions in which impulsivity manifests: decision-making and response inhibition. Activation likelihood estimation (ALE) meta-analyses of task-based fMRI studies within each dimension identified two distinct and non-overlapping functional systems. One located in the default-mode network, associated with value-based judgements and goal-directed decision-making, and the other distributed across higher-order networks associated with cognitive control. Resting-state functional connectivity revealed the two systems were organised into four specialised communities of default-mode, cingulo-insular, fronto-parietal, and temporal regions. Finally, given the widespread use of neurotransmitter-acting medication to treat conditions with impulsive symptoms, we investigated the association between this organisation and neurochemistry and found that integration across communities was associated with PET-derived serotonin receptor density. Our findings reinforce insights from previous behavioural research and provide substantial evidence for the multi-dimensional nature of impulsivity on the neural level. This highlights the necessity for a comprehensive dimensional ontology on all levels of investigation to address impulsivity in a transdiagnostic manner.

## 1 Introduction

Impulsivity has been defined as a tendency toward acting rapidly and/or with diminished forethought or consideration of negative consequences to oneself or others (Dalley & Robbins, 2017;Hamilton, Littlefield, et al., 2015). Impulsive behaviours are a pervasive part of life for many individuals, from reckless driving (Teese & Bradley, 2008) or reactive aggression (Gvion & Apter, 2011) to smoking (Sharma et al., 2014) or thrill seeking (Cyders et al., 2007;Whiteside & Lynam, 2001). Thus, impulsivity plays a crucial role in the human condition, being strongly intertwined with cognitive control and decision-making (Dalley et al., 2011). Heightened impulsivity is believed to be a hallmark of several psychiatric disorders such as attention-deficit/hyperactivity disorder (ADHD), substance abuse, and bipolar disorder (Moeller et al., 2001), which has informed theories of impulsivity as a transdiagnostic marker (Amlung et al., 2019;Berlin & Hollander, 2014). Therefore, understanding the neural mechanisms behind impulsivity is of high research and societal value.

Behavioural and theoretical investigations of impulsivity indicate it is a multi-dimensional psychological construct (Bari & Robbins, 2013;Caswell et al., 2015;Dalley & Robbins, 2017;Dalley et al., 2011;Dick et al., 2010;MacKillop et al., 2016;Reynolds et al., 2006,^2008^), and some authors even argue there is no single umbrella construct of impulsivity at all (Cyders, 2015;Strickland & Johnson, 2021). Despite variations in proposed models, suboptimal response inhibition and decision-making concerning delayed consequences frequently appear as key cognitive-behavioural dimensions. Delayed-consequence sensitivity (DCS) reflects the subjective decrease in reward value as a function of the delay in obtaining that reward and is classically investigated using the delay discounting paradigm (Strickland & Johnson, 2021). Impulsive individuals tend to display steeper discounting, which is the tendency to prefer smaller more immediate rewards over larger later ones (Amlung et al., 2019;Frost & McNaughton, 2017). This behaviour has been otherwise labelled as “impulsive choice” (Hamilton, Mitchell, et al., 2015;Winstanley et al., 2006) or “impulsive decision-making” (Sharma et al., 2014). Response inhibition, the capacity to inhibit a prepotent response tendency, is typically investigated using go/nogo, stop-signal, or 5-choice serial reaction time tasks. Its failure has been considered impulsive and has been referred to as “impulsive action” (Winstanley et al., 2006) or “rapid-response impulsivity” (Hamilton, Littlefield, et al., 2015). It is characterised by premature responses resulting in commission errors (not stopping/responding when instructed to) and impaired reaction time (Bari & Robbins, 2013;Lipszyc & Schachar, 2010;Ioannidis et al., 2019). The reaction time on the stop-signal task (SSRT) is traditionally estimated using the independent race model, conceptualised as a function of go reaction time and stop-signal delays (Logan & Cowan, 1984).

Studies investigating the neural mechanisms of impulsivity in humans have mainly focused on investigating associations between self-reported impulsive traits and brain activation derived from response inhibition and DCS tasks (Christakou et al., 2011;DeVito et al., 2013;Sripada et al., 2011;Wilbertz et al., 2014) or other unrelated paradigms (Anandakumar et al., 2018;S. Wang et al., 2017). However, self-report measurements from trait-based or personality models of impulsivity (Cyders et al., 2007;Whiteside & Lynam, 2001) are unrelated to assessments of behavioural performance and provide a largely independent body of evidence (Cyders & Coskunpinar, 2011;Sharma et al., 2014;Strickland & Johnson, 2021). Other studies have instigated brain activation related to impulsive responses on both dimensions without mixing state and trait impulsivity (Boecker et al., 2011;McClure et al., 2004) as well as associations with model-based variables from these tasks, such as the discounting parameter k or subjective valuation of rewards in DCS (Kable & Glimcher, 2007). These studies point to the ventral striatum and ventromedial PFC, sometimes referred to as the valuation system, underlying impulsive choices in DCS (Noda et al., 2020;Owens et al., 2017;Schüller et al., 2019). Conversely, the posterior medial frontal cortex covering the pre-supplementary motor area (pre-SMA) and anterior midcingulate cortex (aMCC) is believed to play an important role in error monitoring when inhibition fails, as activity in these regions has been reliably found during commission errors (Cieslik et al., 2023;Ullsperger et al., 2014).

A large body of theoretical work considers impulsivity as a form of a trade-off between self-control and impulsive responses (Dalley et al., 2011;Sharma et al., 2014;Whiteside & Lynam, 2001). Therefore, a comprehensive account of the neural mechanisms behind impulsivity within each dimension ought to capture regions related to “controlled” responses (i.e., successful inhibition and shallow discounting) on top of those linked to “impulsive” responses (i.e., commission errors and steeper discounting) discussed above. Specifically, evidence suggests that the regions within the multiple-demand network (Duncan, 2010) such as the anterior insula, medial frontal cortex, right fronto-parietal regions, and basal ganglia subserve inhibitory control exerted to prevent premature “go” responses, that is successful inhibition (Cieslik et al., 2023;Hamilton, Littlefield, et al., 2015;Verbruggen & Logan, 2008;R. Zhang et al., 2017). Conversely, the right dorsolateral prefrontal cortex (PFC) and parietal regions have been associated with choices of larger later rewards (LL) and shallower discounting (Hamilton, Mitchell, et al., 2015;Noda et al., 2020;Owens et al., 2017;Schüller et al., 2019). Finally, within the framework of the independent race model (Logan & Cowan, 1984), where performance on the stop-signal task is modelled as a race between go and stop processes, both processes may have independent control mechanisms with dedicated circuits. Studies investigating their neural correlates point to the right inferior frontal cortex subserving the control of stopping (Aron et al., 2014). Taken together, brain activation studies of impulsivity point to two largely distinct functional systems associated with response inhibition and DCS with a potentially overlapping fronto-parietal control system. However, despite extensive research, a comprehensive picture of the topic is still missing as dimensions have been studied predominantly in isolation. Furthermore, systematic comparisons of the functional architecture related to both controlled and impulsive responding across dimensions are lacking.

While brain activation studies of impulsivity point to two largely distinct functional systems, they cannot rule out regional interactions across networks. Consequently, to better understand the functional organisation underlying these networks, it is important to also consider their connectivity. Resting-state functional connectivity has emerged as a valuable tool for evaluating the functional organisation of the brain, suggesting it is organised into large-scale networks that integrate information across spatially distributed regions (van den Heuvel & Hulshoff Pol, 2010;Yeo et al., 2011). Disruptions to this organisation and its efficiency have been linked to cognition and many psychiatric disorders (Bassett et al., 2018;Castellanos et al., 2013;Shine, 2019). Whereas the regions underlying response inhibition and DCS can be generally attributed to large-scale networks based on their spatial overlap, the connectivity between the regions associated with controlled and impulsive responding within and across dimensions, specifically, has not been investigated.

Beyond activation and connectivity on the level of brain regions, neurotransmitter systems play a significant role in many theoretical accounts of impulsivity as mechanisms modulating behavioural performance (Chamberlain & Sahakian, 2007;Dalley & Robbins, 2017;Dalley et al., 2011). Psychostimulant drugs such as methylphenidate, which block the reuptake of dopamine and norepinephrine, can substantially alleviate symptoms and improve response inhibition even in healthy individuals (Aron & Poldrack, 2006;Hanwella et al., 2011;Nagashima et al., 2014). Functionally, these improvements may be partly ascribed to increased right inferior frontal and insula activation (Rubia et al., 2014). Atomoxetine, a norepinephrine reuptake inhibitor, reduces delay discounting and boosts inhibition in rodents (Robinson et al., 2008). Outside psychostimulants, dopamine has been associated with addiction (Berke & Hyman, 2000;Wise & Robble, 2020) and is a major candidate for passing reward prediction errors within the valuation system (Nasser et al., 2017). Findings from the animal literature show that lesions to the nucleus accumbens—a dopamine-rich nucleus—increase impulsivity on DCS tasks and may also impair response inhibition (Basar et al., 2010). Finally, there is some evidence for the involvement of serotonin in response inhibition, which is impaired following serotonin depletion (Worbe et al., 2014). It has also been inversely related to aggression, a behavioural manifestation of impulsivity, with serotonin 5HT1A/1B receptor agonists reducing aggressive behaviour (de Boer & Koolhaas, 2005;da Cunha-Bang & Knudsen, 2021;Duke et al., 2013). On the level of large-scale functional networks, neurotransmitter systems may facilitate flexible behaviour by dynamically modulating the balance between segregation and integration between network regions (Hansen et al., 2022;Shine, 2019). These in turn may be observed as changes in resting-state functional connectivity, manifesting as reshaping of network organisation (Shafiei et al., 2019;Shine et al., 2018).

Here we aimed to comprehensively delineate the brain networks associated with impulsivity using coordinate-based ALE meta-analyses (Eickhoff et al., 2009,^2012^;Turkeltaub et al., 2002) and resting-state functional connectivity. We focused on two cognitive-behavioural dimensions that show consensus across most performance-based models of impulsivity and are commonly investigated with neuroimaging: delayed-consequence sensitivity and response inhibition. We mainly meta-analysed both the activity associated with “impulsive” responding (i.e., impulsive action and choice) characterised by commission errors or preference for SS rewards and non-impulsive, “controlled” responding characterised by successful inhibition or preference for LL rewards within each dimension. Furthermore, we searched the literature for brain activation associated with model-based performance metrics that are well established for modelling task performance in behavioural literature: discounting factor k, subjective valuation of reward in the delay discounting task (Kable & Glimcher, 2007), and SSRT in the stop-signal task (Verbruggen & Logan, 2008). Next, we characterised the functional network organisation using connectivity and graph-theoretical methods, in two independent large-scale datasets, and investigated whether the two dimensions are subserved by distinct functional networks. Finally, given the widespread use of neurotransmitter-acting medication to treat conditions with impulsive symptoms (Chamberlain & Sahakian, 2007), we investigated in a follow-up exploratory analysis the associations between network organisation (measured as integration and segregation) and receptor density. We restricted these analyses to neurotransmitter systems (dopamine, serotonin, and norepinephrine) that have been functionally and genetically associated with impulsivity (Dalley et al., 2011).

## 2 Methods

### 2.1 Meta-analysis

We performed a literature search using PubMed (https://www.ncbi.nlm.nih.gov/pubmed) and Web of Science (https://webofknowledge.com) for articles published until the 10th March 2022 that investigated brain activation related to either a DCS or response inhibition with fMRI or PET. Additionally, reference tracing of systematic reviews and meta-analyses (on the topics of impulsivity more broadly), as well as response inhibition and delay discounting was done. The search terms were selected in keeping with the “pure measures” of impulsivity within each dimension as suggested byStrickland and Johnson (2021). For DCS, these were “delay discounting,” “temporal discounting,” and “delayed reward” as well as each of the keywords separately. The database for response inhibition studies using the go/nogo and stop-signal paradigms in adults was obtained from a recent meta-analysis byCieslik et al. (2023). We enriched this database by adding studies with adolescent participants for which we used the same search terms as presented inCieslik et al. (2023), namely “stop signal task,” “go no-go task,” “go nogo task,” “response inhibition,” “inhibition,” “action withholding,” “action cancellation,” “action inhibition,” “motor inhibition,” and “inhibitory control.”

We included only results from peer-reviewed fMRI or perfusion PET experiments reporting results of whole-brain group analyses as coordinates in a standard neuroanatomical reference space (Talairach/Tournoux or Montreal Neurological Institute). Results from region-of-interest (ROI) analyses and studies with partial brain coverage were excluded. Only data from healthy participants (including healthy control groups from patient studies) with mean age >= 12 (with an absolute minimum age of individual participants no lower than 10) were retained. Studies with pharmacological interventions, connectivity-based analyses, and single-subject reports were excluded. For studies reporting more than one eligible experiment obtained in the same sample, the reported coordinates were pooled to form a single experiment when included in the same meta-analysis (i.e., coordinates from go/nogo and stop-signal tasks in the same subject group were pooled). If each experiment included a different set of participants, coordinates were not pooled. In cases where different studies or experiments reported results from partly overlapping samples such as inKable & Glimcher (2007and^2010^), coordinates were pooled to form a single experiment and the smaller sample size of the two original experiments was used as the input to the analysis. In cases where any of the above criteria were unclear from screened publications, the corresponding authors were contacted. Lastly, authors of clinical studies that passed our inclusion criteria but reported pooled activation for clinical and healthy control groups were contacted for data from the healthy control group only. Of these, three authors responded and are indicated in the table of included studies in theSupplementary Material. For a reporting checklist detailing analysis and study selection choices as suggested byMüller et al. (2018), seeSupplementary Table S1.

Our contrasts of interest were, in general, analyses contrasting impulsive with non-impulsive, “controlled” behaviour and vice versa, as impulsivity in the pertinent paradigms is behaviourally expressed by a higher frequency of “impulsive responding” such as commission errors (failure to inhibit action when necessary) or choices of smaller but sooner rewards (over larger but later ones). To differentiate the two types of contrasts, we refer to contrasts reflecting impulsive behaviour as “impulsive responding” and to the reverse contrasts reflecting non-impulsive behaviour as “controlled responding.” Secondly, we searched for correlations with model-based variables extracted from behaviour (discounting parameter k, subjective value and SSRT). While for DCS there was a sufficient number of studies reporting model-based variables (especially subjective value), we only found one study investigating SSRT that fulfilled our inclusion criteria. We therefore did not consider SSRT for further analyses. Experiments reporting relative deactivations were interpreted as results of the opposite contrast to that specified (e.g., deactivation observed in a smaller sooner > larger later rewards contrast was interpreted as activation associated with larger later > smaller sooner rewards) unless otherwise specified in the respective publication. A detailed description of the selected contrasts for each impulsivity dimension is provided below. After the exclusion of unsuitable studies (seeFig. 1), the final sample consisted of 46 studies reporting 72 experiments on delayed-consequence sensitivity (18 reporting impulsive responding, 26 controlled responding, 4 correlation with discount factor*k*, and 24 correlation with subjective value) and 100 studies reporting 123 experiments on response inhibition (26 reporting impulsive responding, 96 controlled responding, and 1 correlation with SSRT). Details on all studies included can be found in theSupplementary Material.

**Fig. 1.**
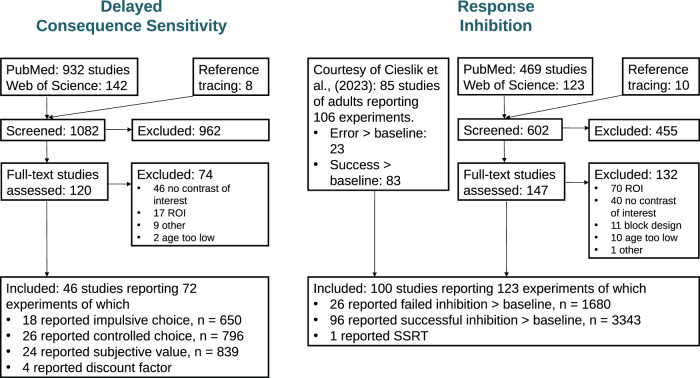
Flow diagram of study selection for the meta-analyses. The total sample size for each contrast is denoted with n. In the flow diagram, “study” refers to a publication and “experiment” to the specific contrast reported. In case a study reported multiple contrasts within the same category (e.g., stop-signal), it was counted as one experiment.

#### 2.1.1 Delayed-consequence sensitivity

Experiments were separated into three categories: impulsive responding, controlled responding, and subjective value and separate meta-analyses were calculated for each category. For impulsive responding, results of smaller sooner (SS) > larger later (LL) rewards, immediate > delayed choice, and*β*>*δ*contrasts were selected, while for controlled responding, the opposite contrasts were included, namely LL > SS, delay > immediate, and*δ*>*β*.*β*is theorised to reflect an “impatient system” and is usually coded in fMRI paradigms as blocks of trials where immediate rewards are possible, while*δ*represents the “patient system” and is coded as blocks of choices where only delayed choices occur (Laibson, 1997;McClure et al., 2004). We further included contrasts that tested for across-participant correlations between brain activity and the temporal discount parameter*k*(or similar constructs reflecting the degree to which individuals discount future rewards). However, as we could not identify enough experiments correlating whole-brain activation with k (three experiments reported positive correlations and one reported negative correlations) for calculating a separate analysis, we have included these experiments in our meta-analyses of impulsive and controlled responding. As higher*k*indicates stronger impulsive tendencies, positive correlations were included in the meta-analysis of impulsive responding and negative correlations in the analysis of controlled responding. Lastly, the parametric modulation and correlation of activity with subjective value were coded as a third category of experiments as choices between SS and LL rewards are highly influenced by the perceived subjective value of the rewards, which is believed to track the valuation processes during delay discounting tasks (Kable & Glimcher, 2007;Schüller et al., 2019). Therefore, in total we included 46 studies reporting 72 experiments on DCS (21 reporting impulsive, 27 controlled responding, and 24 subjective value).

#### 2.1.2 Response inhibition

Similarly to DCS, we aimed to include three categories of studies: impulsive responding, controlled responding, and model-based variables. Following the guidelines for performing well-powered fMRI meta-analyses (Eickhoff et al., 2016;Müller et al., 2018), we were not able to find a suitable number of experiments reporting results of the direct comparison between impulsive and controlled responding in stop-signal and go/nogo tasks (with only 15 for impulsive > controlled and 7 experiments for controlled > impulsive). We, therefore, selected experiments contrasting against control conditions not reflecting impulsivity like “Go” conditions (no need for inhibition) or rest/fixation and then calculated the contrast of interest (impulsive vs. controlled) on the meta-analytical level. In particular, experiments that contrasted brain activation during commission errors or successful inhibition against baseline (Go, fixation or rest) were included. First, we calculated separate meta-analyses for impulsive responding > baseline and controlled responding > baseline, respectively. Next, we compared impulsive and controlled responding by calculating meta-analytic contrasts and conjunction analyses (for further details, see section Activation Likelihood Estimation below). Regions found in the conjunction thus reflect control signals found for both, successful and failed Stop, while those identified with the contrast analyses are more associated with one condition than with the other. Finally, separate analyses were calculated for the two tasks (SST and Go/No-Go) separately in order to ensure that results from the overall analyses combining the tasks were not driven by one of them. As mentioned above, within behavioural performance models, impulsivity has been not only operationalised as failures of inhibition but also as the latency of inhibition. Based on this, the stop-signal reaction time (SSRT) is often estimated using the race model (Logan & Cowan, 1984) during the performance of the stop-signal task. Unfortunately, we were not able to identify a suitable number of studies investigating the correlation of activation with SSRT that fulfilled our inclusion criteria. This might be due to the fact that in fMRI the Stop-Event can be modelled and, therefore, the main focus is on contrasts between the Stop and Go (or failure) events. After excluding unsuitable studies (68 ROI only analysis and 22 not reporting our contrast of interest; see the ANIMA database for a full breakdown of this search), only a single study byHu et al. (2014)was identified. We, therefore, decided to only focus on the contrast between conditions for response inhibition.

#### 2.1.3 Activation likelihood estimation (ALE)

All meta-analyses were performed using the ALE algorithm for coordinate-based meta-analysis of neuroimaging studies (Eickhoff et al., 2009,^2012^;Turkeltaub et al., 2002) that was implemented using in-house Matlab (version 2017a) tools. The analyses were executed as described previously (Kogler et al., 2020) and according to the best-practice guidelines for neuroimaging meta-analyses (Müller et al., 2018). The ALE algorithm aims to identify brain areas where activity across many experiments converges more strongly than would be expected from a random spatial association. Briefly, to reflect the spatial uncertainty of activations, each activation focus was modelled as a centre of a 3D Gaussian probability distribution based on empirical data of between-template and between-subject variance. The between-subject variance was weighted by the number of participants in the respective experiment. For a given experiment, the probability distributions of each focus were then combined and a union over all experiments’ activation maps was computed. This yielded a voxel-wise estimated activation likelihood map (i.e., a map of ALE scores), which describes the degree of spatial convergence across all experiments. Lastly, in order to identify “true” convergence, the ALE scores were compared with an analytically derived null distribution (Eickhoff et al., 2012) reflecting random spatial associations between activation maps for all experiments. Results were thresholded at p < .05 (family-wise error-corrected at cluster level with voxel-level cluster inclusion threshold at p < .001;Eickhoff et al., 2016).

#### 2.1.4 Meta-analytic contrast and conjunction analyses

Contrast and conjunction analyses were calculated between meta-analytic results within each behavioural dimension (i.e., for impulsive vs. controlled) to directly compare impulsive and controlled responding for response inhibition and simplify peak extraction (see below). Commonalities between the meta-analyses (both within-dimension and between dimensions to evaluate overlapping regions) were assessed via conjunction analysis, which identifies voxels with significant convergence in both meta-analyses, calculated as the intersection of the cFWE-thresholded result maps. A cluster extent threshold of at least five voxels was applied to the resulting conjunction maps.

For contrast analyses, the voxel-wise differences between ALE scores of two meta-analyses were calculated and compared with a null distribution of difference scores. This null distribution was derived by pooling all experiments from the two meta-analyses and randomly dividing them into two groups of the same sample size as the original sets. This procedure was repeated 25,000 times to yield an empirical null distribution of ALE-score differences which the observed difference in ALE scores was tested against. The resulting voxel-wise non-parametric p values were thresholded at p < 0.05, with a cluster-wise extent threshold of at least five voxels. While for delayed-consequence sensitivity the number of included experiments was quite similar, for response inhibition, the meta-analyses of controlled versus impulsive responding were unbalanced (96 vs. 26 experiments). To accommodate for the higher power of the controlled responding > baseline meta-analysis, we employed a subsampling procedure described in detail in theSupplementary Methods.

#### 2.1.5 Peak extraction

Next, we created a network comprising all regions involved in response inhibition (impulsive and controlled responding) and DCS (impulsive responding and subjective value). We thus combined the peaks of all meta-analytical networks into one single network. Peaks were extracted from the conjunction and contrast analyses between the meta-analyses of each behavioural task dimension (as described above). Thus, the peaks were on the one hand based on those regions that were found to be involved in more than one meta-analysis as well as those that showed stronger convergence in one compared with another meta-analysis (within the DCS and response inhibition dimension, respectively). Peaks that lay in grey matter were thus extracted from the respective conjunction and contrast maps using fsl5 (Smith et al., 2004) [cluster] command with the minimum distance between peaks set to 15 mm. For peaks coming from different maps (for example, conjunction and contrast maps) that were less than 15 mm apart from each other, we included only the peak with the higher z-score (Nostro et al., 2018). The extracted meta-analytic nodes and all result maps are available in the ANIMA database (Reid et al., 2016):https://anima.fz-juelich.de/studies

### 2.2 Follow-up connectivity analyses for network characterisation

#### 2.2.1 Participants

For all connectivity modelling, two different datasets were used: one served as the discovery sample and one for replicating results. The discovery sample was chosen based on its cross-sectional design reflecting the sample used in the meta-analysis. This allowed us to use the same cut-off age range of 10 to 75 years present in the meta-analysis. Written consent from all subjects and ethics approval were obtained locally at both sites. A joint re-analysis of the anonymised data was approved by the ethics committee of the Heinrich Heine University Düsseldorf (study ID: 2018-317-RetroDEuA).

As a discovery sample, we used the extended Nathan Kline Institute Rockland dataset (Nooner et al., 2012). This amounted to resting-state and anatomical (f)MRI data of 608 healthy subjects (395 female) aged 10–75 years. Only data from participants who had completed the full 10 min of scanning without excessive movement (defined here as mean frame-wise displacement of ≤0.5 mm) were included in further analyses, resulting in a final sample of n = 528 healthy subjects (338 female, age: 10–75 years). We used whole-brain T1 anatomical MPRAGE images (TR = 1,900 ms; 1 mm isotropic voxels) and resting-state fMRI (rsfMRI) multi-band echo-planar imaging (EPI) scans (TR = 1,400 ms; 2 mm isotropic voxels; duration = 10 min; 440 volumes), acquired on a 3-T Siemens Magnetom scanner.

For replication, the minimally pre-processed data of a sample of unrelated healthy subjects (n = 339, 184 female, aged 22–35 years) were obtained from the full release of the Human Connectome Project dataset (Van Essen et al., 2013). We excluded participants with incomplete resting-state scans or excessive movement (mean frame-wise displacement of >0.2 mm as used previously by, e.g.,Yang et al., 2016) resulting in a final sample of n = 336 subjects (183 females, age: 22–35 years). The rsfMRI HCP scanning protocol involved acquiring whole-brain multi-band gradient-echo EPI volumes on a 3-T Siemens “Connectome Skyra” scanner (TR = 720 ms, 2 mm isotropic voxels). Four rsfMRI sessions with 1,200 volumes in total (14 min and 24 s) were acquired over two consecutive days, with one left-to-right (LR) and one right-to-left (RL) encoding direction acquired on each day. For the purposes of replicating our findings based on the eNKI sample, only data from the first session on the first day were used (so-called rest1LR).

#### 2.2.2 Preprocessing

The eNKI data were pre-processed using fMRIPrep version 20.1.1 (Esteban et al., 2019; fMRIPrep 2020), which is based on Nipype version 1.5.0 (Gorgolewski et al., 2011; Nipype 2017). For a detailed description of each step, seeSupplementary Methods. Briefly, this included skull-stripping, head-motion correction, and slice-time correction. The BOLD images were then co-registered to the native space of the subjects’ T1w image, normalised to MNI space, and motion-corrected.

The HCP data used here were minimally pre-processed. The pre-processing pipeline has been described in detail elsewhere (Glasser et al., 2016). Briefly, this included gradient distortion correction, image distortion correction, registration to subjects’ T1w image and to MNI standard space followed by intensity normalisation of the acquired rsfMRI images, and ICA FIX denoising (Salimi-Khorshidi et al., 2014).

For both datasets, additional denoising steps were undertaken using fMRIPrep output files or data provided by the HCP and in-house scripts in MATLAB (version 2019b). First, we regressed mean time courses of two tissue classes (white matter and cerebrospinal fluid) and the global signal which has been shown to reduce motion-related artefacts (Ciric et al., 2017). Next, data were linearly detrended, bandpass-filtered at 0.01–0.1 Hz, and spatially smoothed using a Gaussian kernel of FWHM = 5 mm.

#### 2.2.3 Community detection and network measures

After averaging the time series from all grey-matter voxels within 5-mm spheres around the meta-analytically derived coordinates, node-to-node functional connectivity was calculated as the Pearson correlation between the time courses of each node. The resulting connectivity matrix for each participant was z-scored using Fisher’s z transformation and averaged across all participants. We employed the Louvain algorithm (Blondel et al., 2008), a stochastic method, for identifying distinct communities within a network by optimising Q, a modularity score (Betzel, 2020). For this, we used the community_louvain.m function from the Matlab-based Brain Connectivity Toolbox (Rubinov & Sporns, 2010). The averaged connectivity matrix between all meta-analytic nodes was used as the input. We fine-tuned the community assignment by using the communities resulting from applying the algorithm to the connectivity matrix as an additional input and repeated the procedure until Q remained constant. Given the greedy stochastic nature of the algorithm (Good et al., 2010), community assignment was evaluated by repeating the procedure 1,000 times to obtain an agreement matrix. To evaluate the node roles in the final community partition, we calculated the participation coefficient (participation_coef_sign.m) and within-module degree z-score (module_degree_zscore.m). The participation coefficient identifies whether a node’s connections are distributed across communities or clustered within a community and reflects between-module integration at high values and segregation at low values. The within-module degree z-score describes the connectedness of a node to its own community relative to other nodes in the same community and thus reflects within-module integration.

#### 2.2.4 Seed-voxel connectivity gradients

While the above-described community detection identifies communities based on node-to-node connectivity profiles, we additionally investigated network organisation based on the “node-to-rest of the brain” connectivity profiles (i.e., seed-to-voxel correlations). In order to determine whether nodes located in the same community displayed similar connectivity to the rest of the brain, we identified principal axes of variation in the connectivity profiles across all nodes. This technique was recently used to determine spatial variation in both node-to-node (Margulies et al., 2016) and seed-to-voxel (J. Zhang et al., 2019) connectivity, as well as structural characteristics such as microstructure (Paquola et al., 2019) across the cortex. Seed-to-voxel connectivity was calculated as Pearson correlation between the mean time courses of each node and all remaining grey-matter voxels in the brain, resulting in one connectivity map for each node per subject. Maps for each node were Fisher Z-transformed before averaging across participants. Next, we constructed a node-by-node similarity matrix, by transforming the averaged (3D) seed-to-voxel connectivity map of each node into a vector and correlating the resulting vectors from each node (resulting in a 21 x 21 matrix). To this matrix, we then applied principal component analysis using the BrainSpace toolbox (Vos de Wael). Only the top 20% of node similarities were retained (i.e., sparsity parameter). The remaining parameters were kept the same as in previous work byMargulies et al. (2016), with α set to 0.05. We repeated the gradient decomposition using diffusion map embedding (Coifman et al., 2005) and varying levels of sparsity (30% and 40%) in order to confirm our results were not subject to the choice of dimensionality reduction algorithm or parameters.

### 2.3 PET-based receptor density analysis

To investigate the relationship between neurotransmitter receptor/transporter density and community organisation, we used PET-derived whole-brain maps available in the JuSpace toolbox (Dukart et al., 2021) available online (https://www.fz-juelich.de/inm/inm-7/EN/Resources/_doc/JuSpace.html?nn=2463520). For the analysis, we only used receptor and transporter maps for neurotransmitters theoretically related to impulsivity: serotonin, norepinephrine, and dopamine (Chamberlain & Sahakian, 2007;Dalley & Robbins, 2017;Dalley et al., 2011). In particular, for serotonin, we utilised the 5HT1a, 5HT1b, 5HT2a, and serotonin transporter maps (SERT) (Savli et al., 2012), norepinephrine transporter map NAT (Hesse et al., 2017) for norepinephrine, and D1 (Kaller et al., 2017), D2 (Alakurtti et al., 2015), and dopamine transporter maps (Dukart et al., 2018) for dopamine. All PET maps were acquired from healthy volunteers and rescaled to a minimum of 0 and a maximum of 100; for further details, seeDukart et al. (2021).

First, all the above PET maps were resampled from 3 mm isotropic voxels to 2 mm isotropic voxels using the fsl5 [flirt] command. For each node, we then averaged the receptor density values in all grey-matter voxels within 5 mm diameter spheres around each coordinate. Next, the node-wise receptor density was correlated (using Spearman rank correlation) with within-module degree z-score and participation coefficient derived from the community organisation. Correlations that displayed at least moderate effect size (>+-0.3) in both our discovery and replication datasets were then tested against a spatially informed null model for significance using permutation testing. To this end, we created 1,000 random networks by randomly sampling coordinates from a conservative grey-matter mask. To mirror the spatial properties of our impulsivity network in the randomly sampled networks, we restricted the minimum, mean, and maximum Euclidean distance between the sampled nodes to be within 1 standard deviation from the impulsivity network’s minimum, mean, and maximum values, respectively. We then calculated the Spearman rank correlation between receptor density in nodes of each random network and our empirically derived measures of integration and segregation to estimate a null distribution. The empirical rank correlation was then compared with the estimated null. Correlation coefficients higher than 95% of the random correlations were interpreted as significant. Scripts used for generating random networks are available athttps://github.com/MartinGell/random_nets

## 3 Results

### 3.1 Meta-analysis

Based on our search criteria, we identified 46 studies reporting 72 experiments on DCS and 100 studies reporting 123 experiments on response inhibition. Within DCS, 18 experiments reported impulsive responding (of which 15 were SS > LL, and 3 “impatient system”*β*> “patient system”*δ*) and 26 reported controlled responding (of which 23 were LL > SS, and 3 “patient system”*δ*> “impatient system” β). In total, 24 experiments reported correlation with (or parametric modulation of) subjective value. Only 4 experiments reported a correlation with the discounting parameter k and were, therefore, included in the impulsive and controlled responding meta-analyses (see methods for details). Within response inhibition, we identified 26 experiments that reported activation during failed inhibition against baseline/go and 96 experiments that reported successful inhibition against baseline/go. Finally, only one study reported whole-brain correlations with SSRT and was, therefore, excluded from further analyses.

#### 3.1.1 Delayed-consequence sensitivity

**Fig. 2.**
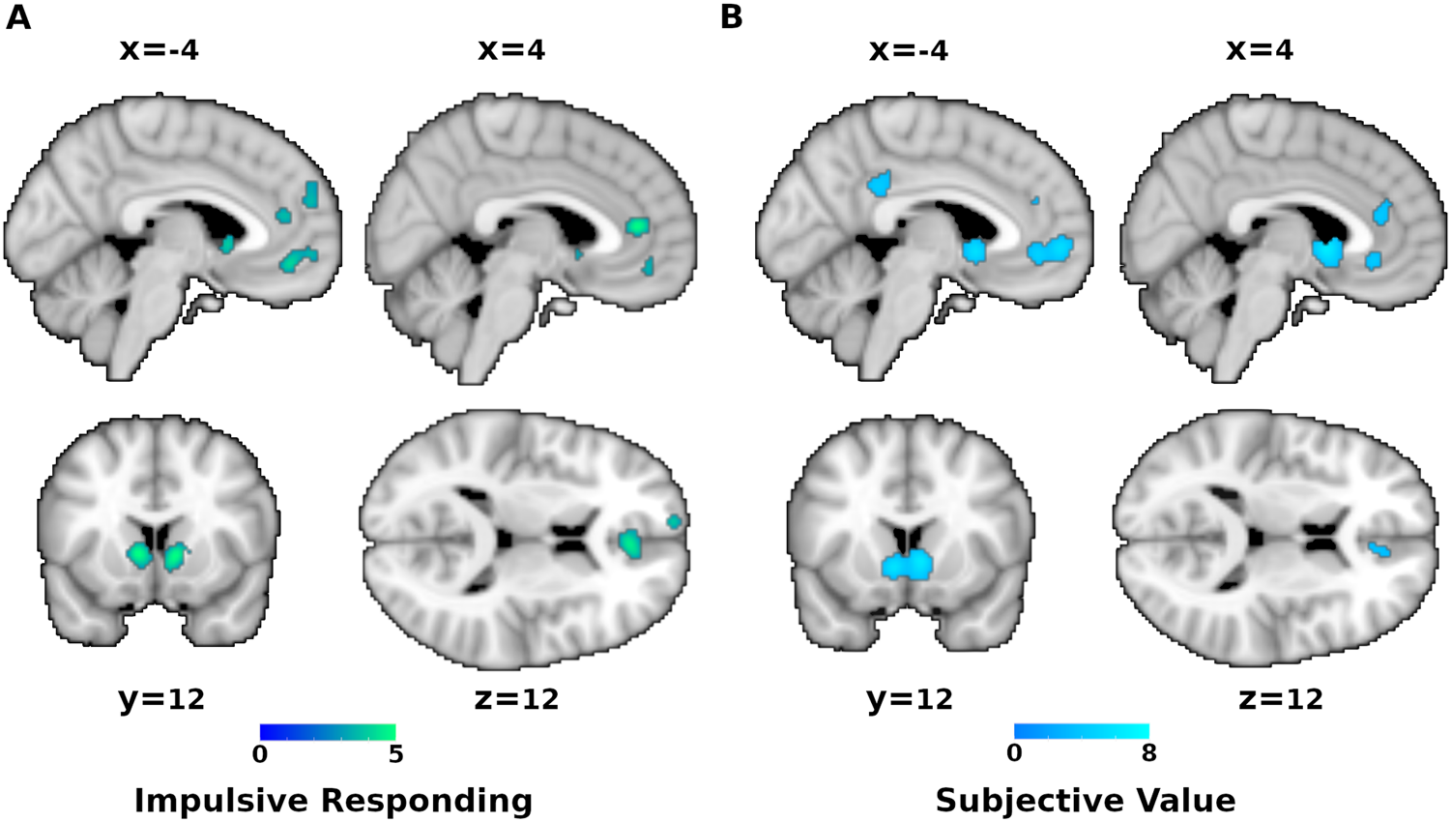
Delayed-consequence sensitivity. Results of the meta-analysis on brain activity correlates of (A) impulsive responding (i.e., preference for smaller sooner rewards) and (B) subjective value. Colour codes z-score.

Analysis of experiments investigating DCS revealed significant findings only for impulsive responding and subjective value contrasts. Impulsive responding (Fig. 2A) led to consistent activation of the ventromedial prefrontal cortex (VMPFC), left frontal pole (FP), ACC, and bilateral ventral caudate extending to the nucleus accumbens hereafter referred to as ventral striatum (VS) (Haber, 2011). Analysis of experiments correlating activity with subjective value revealed convergence in a largely overlapping network (Fig. 2B). Conjunction analysis revealed that left VMPFC, bilateral VS, and right ACC were common in both meta-analyses. Conversely, contrast analyses showed that only FP was specific to impulsive responding, while subcallosal cingulate cortex (scACC) and posterior cingulate cortex (PCC) were specific to subjective value (Supplementary Figs. S1 and S2). There were no converging clusters for experiments testing controlled responding (choices of LL over SS). The exclusion of studies that correlated measures of impulsivity such as the discount rate k (thus including only the “pure” SS > LL and LL > SS contrasts) revealed similar results (Supplementary Fig. S3).

#### 3.1.2 Response inhibition

**Fig. 3.**
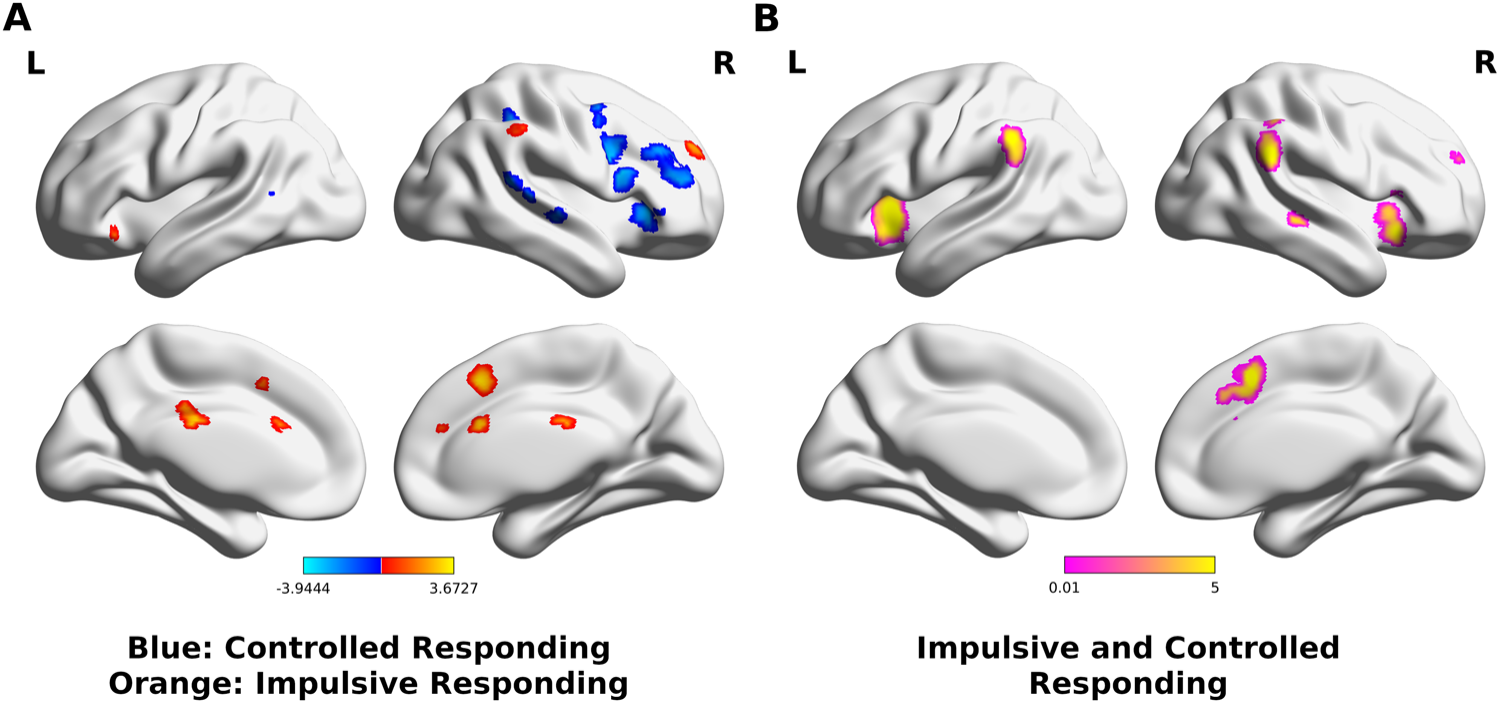
Response Inhibition. Results of (A) meta-analytic contrast and (B) conjunction analyses of successful inhibition > baseline/go and failure of inhibition > baseline/go contrast meta-analyses.

**Fig. 4.**
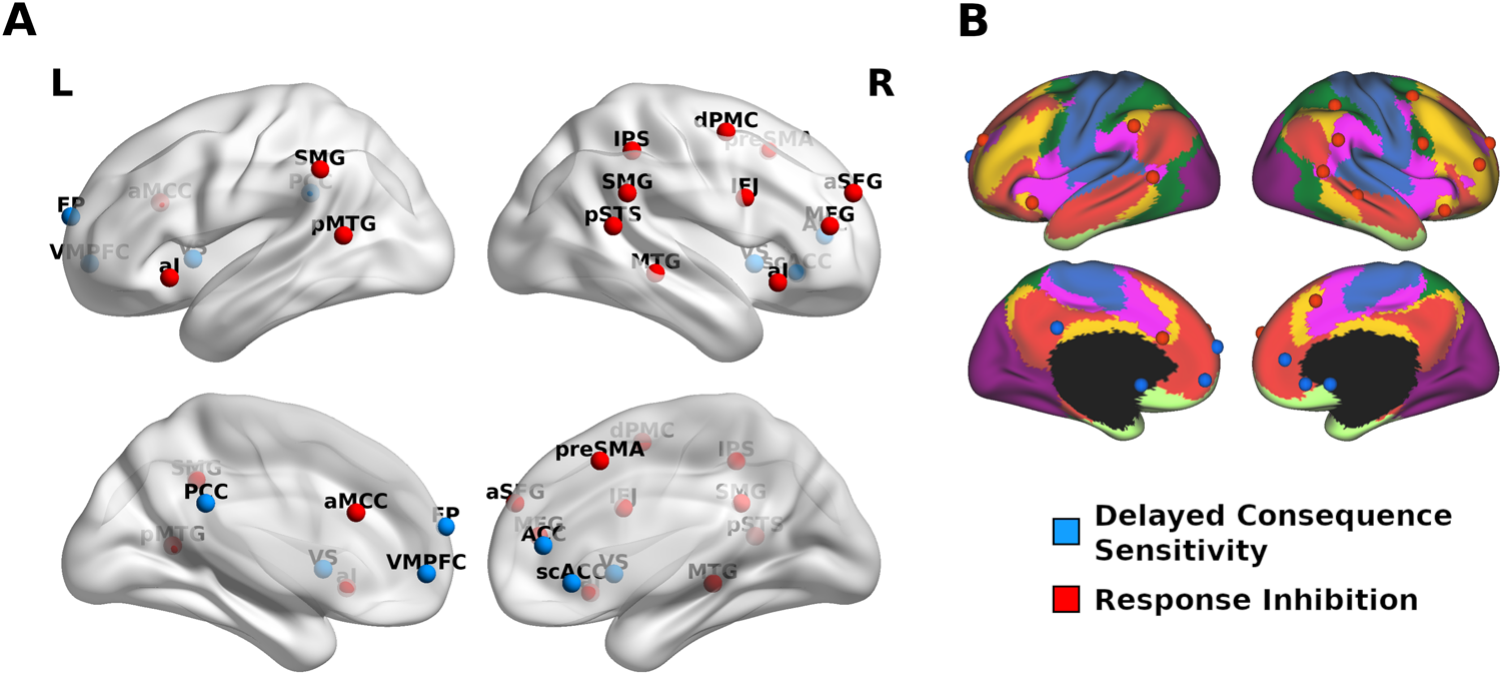
Nodes of the Impulsivity Network. (A) Impulsivity network nodes: Delayed-consequence sensitivity in blue and response inhibition in red. Panel (B) displays impulsivity network nodes overlaid overYeo et al. (2011)resting-state networks: visual (purple), somatomotor (blue), dorsal attention (green), ventral attention (pink), limbic (white), fronto-parietal (orange), and default mode (red) networks.

Results of the first-level meta-analyses of failed or successful inhibition against go and baseline used for meta-analytical contrast of failed against successful inhibition can be found inSupplementary Figure S4andFigure 4. These analyses revealed a widespread network of insular, fronto-parietal, and subcortical regions in line with previous findings (Cieslik et al., 2023). Additionally, we calculated separate meta-analyses for go/no-go and stop-signal tasks, respectively, that showed highly similar results indicating that findings were not driven by one task (Supplementary Figure S4;Fig. 4). The meta-analytic contrast analysis of impulsive responding (failed vs. successful inhibition) revealed stronger convergence in preSMA, aMCC, the right anterior section of the superior frontal gyrus (aSFG), and right supramarginal gyrus (SMG) (Fig. 3A, orange-yellow). Stronger convergence for controlled responding (successful vs. failed inhibition) was found across the lateral frontal and dorsal premotor cortex (dPMC) in addition to the right temporal and parietal regions, right anterior insula (aI), and left putamen (Fig. 3A, blue).Figure 2Billustrates the conjunction analysis across the meta-analyses of failed and successful inhibition against baseline.

### 3.2 Network characterisation

To explore the functional organisation of the resulting meta-analytic networks, we first investigated potential overlap by calculating minimal conjunction and found no overlapping regions. Next, we investigated whether the response inhibition and delayed-consequence sensitivity networks were functionally related by exploring their functional connectivity profiles and community structure. The nodes that were used for these analyses are displayed inFigure 4. For MNI coordinates and complete regions labels (seeTable 1). An overlay withYeo et al.’s (2011)resting-state networks (Fig. 4B) shows that nodes from the DCS meta-analyses were primarily located within medial DMN. Combined controlled and impulsive responding nodes were mostly found in the dorsal attention network, while the remaining nodes were distributed over fronto-parietal and ventral attention networks. The extracted meta-analytic nodes and all result maps are available in the ANIMA database:https://anima.fz-juelich.de/studies(Reid et al., 2016).

**Table 1.** Meta-analytic nodes.

Region (abbreviation)	Hemisphere	MNI coordinate		
		x	y	z
Ventral striatum (VS)	Left	-8	10	0
Ventromedial prefrontal cortex (VMPFC)	Left	-4	44	-8
Ventral striatum (VS)	Right	10	12	-2
Anterior cingulate cortex (ACC)	Right	8	42	10
Frontal pole (FP)	Left	-10	62	18
Subcallosal cingulate cortex (scACC)		2	30	-6
Posterior cingulate cortex (PCC)		-2	-38	28
Anterior insula (AI)	Left	-38	20	-8
Anterior insula (AI)	Right	32	22	-10
Pre-supplementary motor area (preSMA)	Right	4	18	48
Supramarginal gyrus (SMG)	Right	60	-42	28
Supramarginal gyrus (SMG)	Left	-60	-44	36
Middle temporal gyrus (MTG)	Right	54	-30	-6
Superior frontal gyrus (SFG)	Right	24	54	28
Anterior midcingulate cortex (aMCC)		0	24	24
Inferior frontal junction (IFJ)	Right	48	8	26
Middle frontal gyrus (MFG)	Right	40	44	14
Posterior superior temporal sulcus (pSTS)	Right	58	-48	14
Intraparietal sulcus (IPS)	Right	40	-40	46
Posterior middle temporal gyrus (pMTG)	Left	-58	-52	12
Dorsal premotor cortex (dPMC)	Right	38	0	54

MNI—Montreal Neurological Institute.

#### 3.2.1 Community structure

**Fig. 5.**
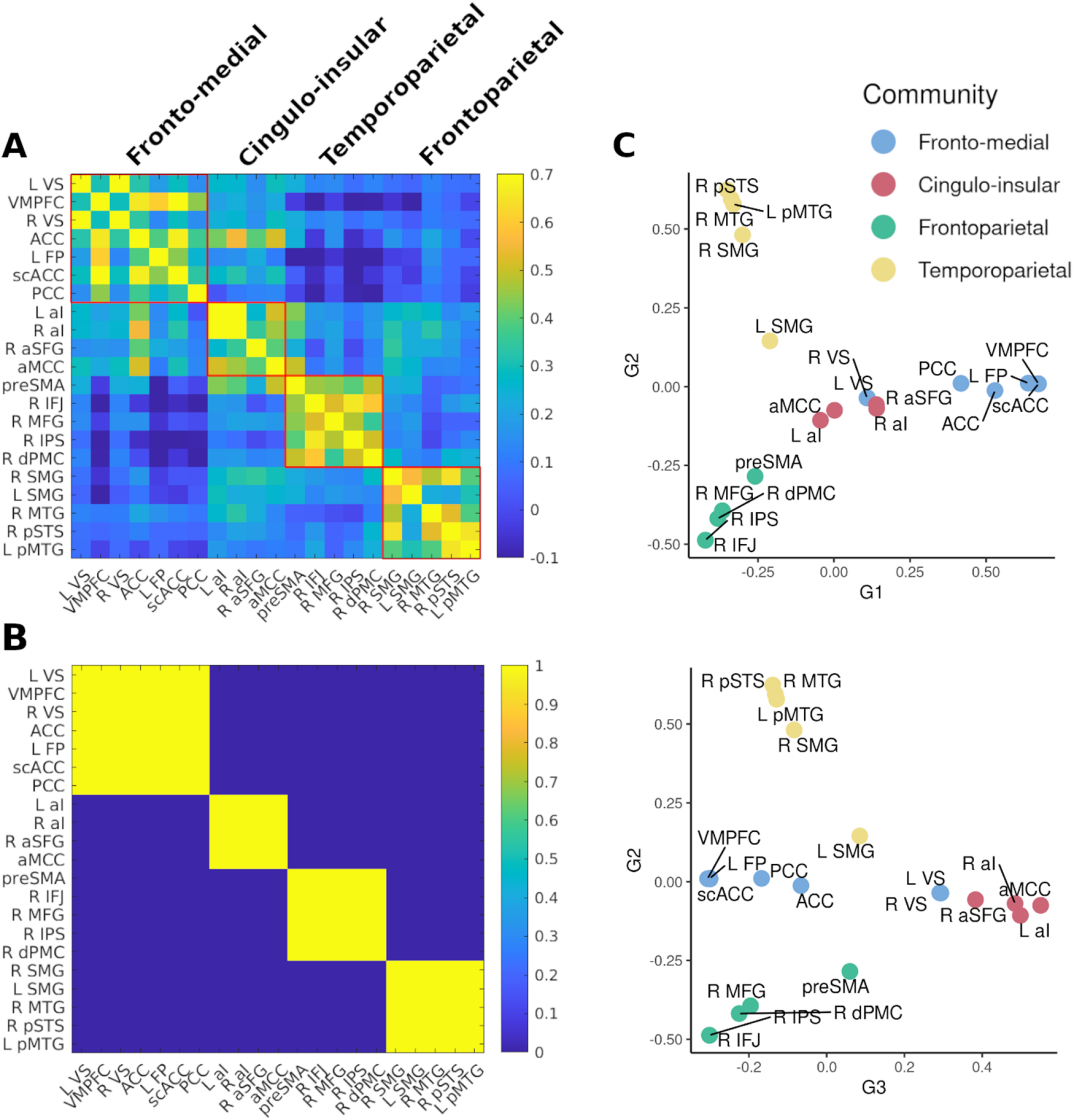
Impulsivity Network Communities. Panel (A) shows connectivity-based communities in the discovery sample (eNKI). The agreement matrix in panel (B) displays the consensus across 1,000 repetitions of the community detection. Legend refers to the proportion of overlapping community solutions. Seed-voxel connectivity gradients are displayed in panel (C). For a 3D depiction of the three components in (C) see:https://github.com/MartinGell/Impulsivity_networks

To detect communities within the impulsivity network we used the Louvain community detection algorithm (Blondel et al., 2008), which divides a network into non-overlapping groups of nodes. Using estimates of resting-state FC between all network nodes from 528 participants of the publicly available Nathan Kline Institute dataset (eNKI) (Nooner et al., 2012) as edges, this approach yielded a four-community solution (Fig. 5A). Repeating this procedure 1,000 times, we observed a strong convergence across solutions suggesting that our four-community solution was not restricted to a local maximum in the solution space (Fig. 5B). To evaluate the robustness of our findings further, we repeated the community detection analysis using a different set of 316 unrelated subjects from the Human Connectome Project dataset (HCP) (Van Essen et al., 2013) and found an identical community structure (Supplementary Fig. S5).

The first fronto-medial community consisted of all DCS nodes in the network (VS, VMPFC, ACC, frontal pole, PCC, and scACC). Regions related to response inhibition were subdivided into three different communities. In the order of appearance inFigure 5, the first of these comprised mostly regions of the so-called salience network (Seeley et al., 2007), that is, bilateral and aMCC as well as right SFG. The next community spanned mainly right-lateralised fronto-parietal regions (IFJ, MFG, dPMC, and IPS) as well as preSMA. The last community consisted of temporo-parietal regions (bilateral SMG, MTG, and pSTS). Interestingly, the cingulo-insular community was the only community to display positive coupling with regions of both the DCS and response inhibition networks.

Finally, we investigated the robustness of the resulting communities by a complementary whole-brain analysis. Here, principal component analysis of the pair-wise similarity between maps of seed-to-voxel connectivity of the meta-analytic nodes was used to explore the dimensions along which they were organised in relation to the rest of the brain (for scree plot seeSupplementary Fig. S6). The initial three components that explained the most variance showed loadings that were in strong agreement with our community detection results, suggesting the node-to-brain interactions paralleled node-to-node relationships (Fig. 5C). The first principal component showed that DCS nodes (except VS) displayed affinity in their connectivity with the rest of the brain while being dissimilar to the response inhibition regions. Similar properties were observed for the cingulo-insular, fronto-parietal, and temporo-parietal communities along the second and third gradient revealing the closeness of within-community nodes in their whole-brain connectivity profiles. Results did not differ with varying sparsity or decomposition parameters.

#### 3.2.2 Network organisation related to receptor density

**Fig. 6.**
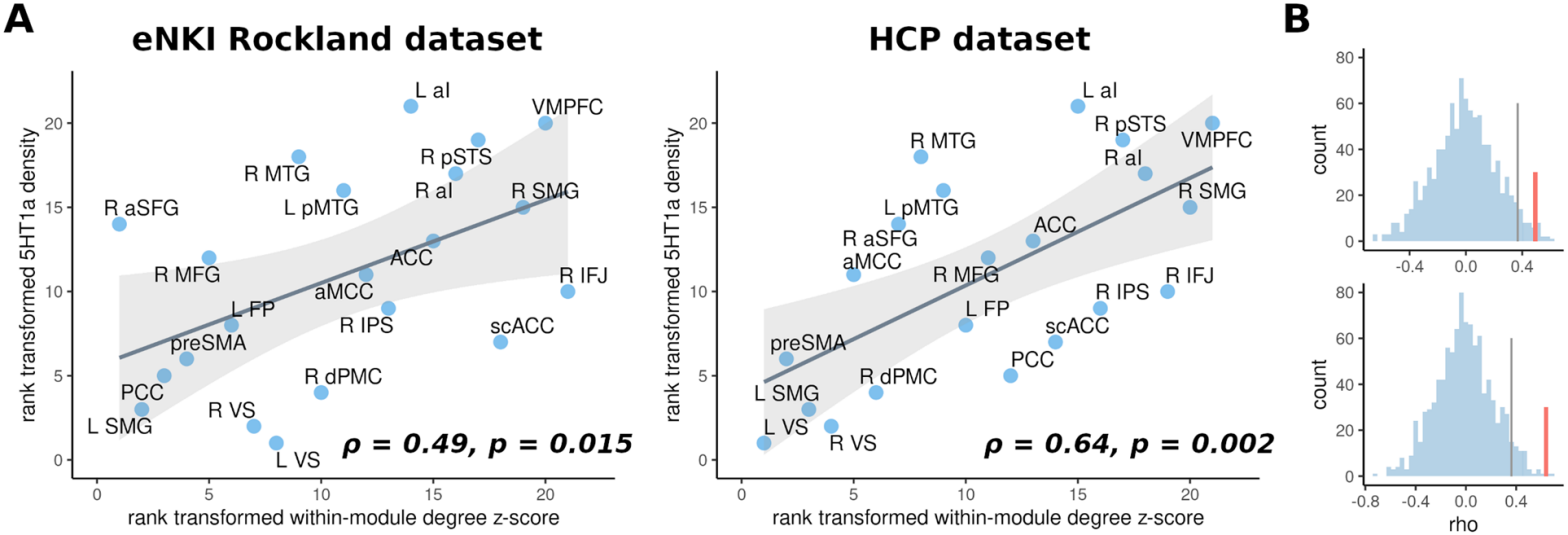
Association of community organisation with serotonin receptor 5HT1a. Panel (A) shows the relationship between within-module degree z-score (high scores indicate within-network integrator regions) and 5HT1a receptor in the discovery (left) and replication sample (right). Panel (B) displays permutation-derived null distributions of correlation coefficients (Spearman’s rho) between receptor density and within-module degree z-score in the discovery sample (top) and replication sample (bottom). Observed correlation is marked with a red line and the significance level of 0.05 is indicated by a grey line.

Finally, we examined whether network organisation was associated with neurotransmitters related to impulsivity across dimensions (Dalley & Robbins, 2017). In particular, given our systems approach, we were interested whether the interactions between network nodes within and between communities are related to dopamine and serotonin receptor density as well as norepinephrine transporter density derived from PET imaging. Network organisation was assessed using two graph-theoretical measures: (i) within-module degree z-score, a measure of how well a node is connected to other nodes in its community, and (ii) participation coefficient, a measure of how well a node is connected to other modules (Guimerà & Nunes Amaral, 2005). Only serotonin 5HT1a receptor density showed a positive relation to within-module degree z-score in both samples (eNKI: ρ = 0.49, p = 0.015; HCP: ρ = 0.64, p = 0.002), suggesting that node-wise serotonin expression was related to within-module integration (Fig. 6).

## 4 Discussion

The present study investigated brain networks associated with two dimensions of impulsivity, response inhibition, and DCS, and provides a comprehensive and fine-grained characterisation of the neural correlates based on task activation, connectivity, and neurochemistry. Using ALE meta-analyses of task-based fMRI studies, we provide evidence for two distinct functional systems: one centred in the medial prefrontal cortex, ventral striatum, and posterior cingulate cortex involved in DCS, and the second covering right lateral frontal cortex, temporo-parietal regions, anterior insula, and anterior midcingulate cortex subserving response inhibition. Community detection based on resting-state functional connectivity between all meta-analytically derived nodes in two large independent samples revealed four functional communities. The fronto-medial community included all DCS regions corroborating their dissociation from the other system. Response inhibition, in turn, was fractionated into three networks spanning fronto-parietal, temporo-parietal, and cingulo-insular regions. Lastly, the integration of individual nodes within those communities calculated in two independent datasets was associated with serotonin receptor density.

### 4.1 Two systems

The results of our meta-analyses indicate that response inhibition and DCS dimensions of impulsivity differ not only in terms of their behavioural manifestations (MacKillop et al., 2016;Sharma et al., 2014;Stahl et al., 2014) but also on the neural level. Mirroring the behavioural dichotomy, we found two distinct sets of regions involved in each dimension. The network of regions associated with DCS was mainly localised within the DMN (Raichle, 2015;Owens et al., 2017), while response inhibition covered the multiple-demand network (Duncan, 2010;Langner et al., 2018;Müller et al., 2015). These findings directly support theoretical accounts proposing separate functional systems for individual impulsivity dimensions (Strickland & Johnson, 2021) and agree with findings on delay discounting (Frost & McNaughton, 2017;Noda et al., 2020) and response inhibition (Cieslik et al., 2023;R. Zhang et al., 2017). The current work now provides a more fine-grained overview by differentiating controlled and impulsive processing within each dimension and considering both in the final network definition. Interestingly, we found no convergence in the dlPFC for the contrast, reflecting a preference for larger later rewards in the DCS meta-analysis that has been posited by previous literature (Hamilton, Mitchell, et al., 2015;Noda et al., 2020;Owens et al., 2017;Schüller et al., 2019) and have, therefore, found no evidence of an overlapping prefrontal control system across the two dimensions. While some individual studies reported activations within this region (e.g.,McClure et al., 2004), a previous meta-analysis bySchüller et al. (2019)reported a convergence across seven studies which is most likely too low to delineate stable results (Müller et al., 2018).

Within the framework of response inhibition, impulsivity has been described as an impairment in executive functioning (with inhibitory control being one of the major executive functions), while from the delayed-gratification perspective, impulsivity has been more associated with motivational processes that underlie decision-making (Bari & Robbins, 2013;Stahl et al., 2014). In line with this, the regions related to DCS identified here, especially vmPFC and ventral striatum, have been previously implicated in value-based decision-making (Haber & Knutson, 2010;Rangel & Hare, 2010). Similarly, regions related to response inhibition have been classically associated with executive functions (Camilleri et al., 2018;Duncan, 2010;Langner et al., 2018). These functional differences echo behavioural findings. Performance on response inhibition and delay discounting tasks is differentially related to treatment outcomes of impulsivity-related disorders (Sheffer et al., 2012;Stevens et al., 2014), impulsive behaviours such as reactive aggression or drug taking (Sharma et al., 2014), and to pharmacological intervention (Worbe et al., 2014;Winstanley et al., 2004). For instance, after reviewing the literature,Stevens et al. (2014)concluded that retention and treatment success in addiction, a condition believed to be strongly related to impulsivity (de Wit, 2009;Dick et al., 2010), were likely related to performance in monetary incentive delay tasks, but not to commission errors in response inhibition. The present findings, therefore, show that behavioural differentiation between the two dimensions is also mirrored on the neural level by the involvement of two distinct neurocognitive systems.

### 4.2 Four communities

Activity within the response inhibition and DCS networks has been linked to both behavioural (Aron & Poldrack, 2006;Hariri et al., 2006;Q. Wang et al., 2016) and clinical (Stevens et al., 2014) variability. However, to develop markers of psychopathology, interactions within and between large-scale systems are essential (Bassett et al., 2018;Castellanos et al., 2013). Moreover, co-occurring deficits in both response inhibition and steeper delay discounting within the same individual in conditions like addiction and ADHD are not uncommon (Bickel et al., 2012;Castellanos-Ryan et al., 2014;Ioannidis et al., 2019;Yücel et al., 2019). Thus, the two networks identified here cannot account for most impulsivity-related variability in isolation. A systems perspective that considers both within- and between-system interactions may be necessary to bridge this gap. To this end, we used resting-state functional connectivity between the meta-analytic nodes as well as between the nodes and the rest of the brain to identify their community organisation based on their intrinsic coupling patterns. Supporting our meta-analytic findings, the fronto-medial community comprised all DCS regions, suggesting tight integration. Conversely, response inhibition regions split into three communities (cingulo-insular, temporo-parietal, and fronto-parietal) that strongly resemble previous reports (Camilleri et al., 2018;Langner et al., 2018).

The fronto-parietal community corresponded to regions within the dorsal attention (IFJ, dPMC, IPS) and fronto-parietal (preSMA, MFG) resting-state networks (Yeo et al., 2011). The dorsal attention network is believed to subserve top-down control of visuospatial attention (Corbetta & Shulman, 2002), including attentional shifting (Kelley et al., 2008), while the preSMA has been implicated in cognitive control (Cole et al., 2013) and motor preparation (Kennerley et al., 2004). Directing attention to expected and relevant stimuli and intentionally enhancing the processing of these stimuli when they occur subserved by the DAN may thus enable the appropriate initiation or inhibition of actions when appropriate (such as when a stop or no-go signal appears). Except for the right MTG (located in the DMN), the temporo-parietal community (bilateral SMG and STS) covered regions located in the posterior ventral attention network. The TPJ, which covers most of the community, has been argued to underlie contextual updating more generally (Geng & Vossel, 2013) and updating responses from action execution to action inhibition during the stop-signal task more specifically (Cieslik et al., 2015). Thus, inefficient updating or transfer of updated information to motor regions via preSMA may result in slower responses or failures of inhibition commonly observed in high-impulsive individuals (Bari & Robbins, 2013).

The last community displayed tight interactions between the anterior insula, aMCC, and aSFG, which have been previously described as the salience network (SN) (Gordon et al., 2017;Seeley et al., 2007). The SN has been associated with detecting important or salient stimuli (Seeley et al., 2007), and is believed to initiate control signals and facilitate switching between higher-order networks (Goulden et al., 2014;Menon & Uddin, 2010). We observed positive associations between the cingulo-insular community and both the fronto-parietal and temporo-parietal communities supporting its role as a control element within the response inhibition network (for a similar account, seeCamillieri et al., 2018). In action inhibition specifically, such top-down signals likely originate from the aMCC which has been previously linked to error monitoring (Ullsperger et al., 2014) and may be crucial to inhibitory planning in the preSMA that displayed a strong association with it. Taken together, by facilitating attention, control, updating, and action planning, the three communities together likely produce the required behaviour: to enact or inhibit an impulsive response tendency.

The cingulo-insular community also displayed a positive association with the DCS subsystem. These results are in line with models of the salience network as a control element mediating the dynamic interactions between DMN and fronto-parietal networks to facilitate goal-directed behaviour (Menon, 2011). Similarly, the cingulo-insular community may play a role in coordinating the fronto-medial and fronto-parietal communities. Aberrant interactions between the fronto-parietal networks, DMN, and SN (i.e., the triple network model) (Menon, 2011) have been proposed to underlie a number of psychiatric disorders. It is thus not unlikely that impulsivity, itself a transdiagnostic marker (Berlin & Hollander, 2014), is related to the functional integrity of the cingulo-insular, fronto-medial, and fronto-parietal communities. Supporting this, connectivity between these large-scale systems has been associated with discounting rate (Chen et al., 2018), ADHD (Cai et al., 2018), addiction (L. Wang et al., 2017;R. Zhang & Volkow, 2019), and impulsive symptoms in Parkinson’s disease (Koh et al., 2020). Similarly, findings of aberrant connectivity between the dlPFC (part of the fronto-parietal subsystem) and ventral striatum (part of the delay sensitivity subsystem) in substance use disorder (Ersche et al., 2020;Jollans et al., 2016) and pathological gambling (Koehler et al., 2013) may be in part explained by a dysfunctional salience control subsystem. As such, inappropriate disengagement of either the fronto-parietal or fronto-medial communities during task execution may result in apparent connectivity changes between them and influence behaviour (Liang et al., 2016;Shine & Poldrack, 2018). Taken together, we propose the multi-dimensional construct of impulsivity is associated with a broad network including default mode, fronto-parietal, temporal, and subcortical regions that can be distinguished into four communities. Interactions between these communities suggest that the entire network is ultimately involved in the final behavioural phenotype of impulsivity.

### 4.3 Neurochemistry

To investigate the biological relevance of the identified community organisation, we explored the relationship between integration and segregation of the impulsivity network with the receptor/transporter density of three impulsivity-related transmitter systems of the brain. These analyses revealed that within-community integrator regions display a higher density of the serotonin 5HT1a receptor, suggesting that integration within communities may be modulated by available serotonin. Evidence of serotonin involvement in different impulsivity dimensions is mixed, with the strongest evidence implicating it in response inhibition (Dalley & Robbins, 2017). There is ample evidence for an inverse association between serotonin levels and aggression, a behavioural manifestation of impulsivity (Carhart-Harris & Nutt, 2017;da Cunha-Bang & Knudsen, 2021;Duke et al., 2013). Specifically, 5HT1A/1B receptor agonists have been shown to reduce aggressive behaviour in many species including humans (Cleare & Bond, 2000;de Boer & Koolhaas, 2005;Popova et al., 2007;Sperry et al., 2003), while reduction in firing has been associated with increased aggression (Audero et al., 2013). Activation within regions that exhibited high within-community integration like the anterior insula and medial PFC has been previously proposed to regulate aggression (Blair et al., 2021). The present findings, therefore, indicate that serotonergic modulation of behaviours such as aggression might be associated with facilitated integration within communities. Interestingly, neither the norepinephrine transporter nor dopamine receptor density was found to be related to functional network organisation. Our results thus indicate that the mechanism of action of norepinephrine and dopamine on function may not be through altering network integration or segregation, warranting further investigation.

### 4.4 Limitations and outlook

Importantly, the present investigation could only evaluate brain networks related to response inhibition conceptualised as the contrast between failed and successful stopping and DCS as the contrast between choices of rewards. We were not able to identify enough studies to independently evaluate associations between activation and the discounting parameter k or SSRT, which have been used as operationalisation of impulsivity in non-imaging studies (Ioannidis et al., 2019). While neuroimaging studies of brain–behaviour relationships with k are in general rare, associations with SSRT are typically investigated with regions of interest approach, which is unsuitable for ALE meta-analyses (Müller et al., 2018). Given that regions of interest were often selected from significant clusters of activations in the specific contrasts that we meta-analysed, such as successful inhibition versus go (Manza et al., 2016;Osada et al., 2019;Xu et al., 2022), the networks reported in our meta-analyses will mostly overlap with regions commonly showing specific correlation with SSRT. Furthermore, evidence from studies using electrocorticography that has a higher spatio-temporal resolution than fMRI suggests that activity in the right inferior frontal cortex is crucial for both the process of stopping and its latency (Aron et al., 2014). The inferior frontal cortex was a locus of multiple clusters of convergence across studies in our successful inhibition versus go meta-analysis, thus suggesting the response inhibition networks identified here may also support latency of stopping. However, in order to facilitate more comprehensive meta-analyses of the processes underlying response inhibition and DCS and in particular impulsivity, future work ought to report (or provide) also results that are not the primary focus of interest (e.g., whole-brain contrasts when investigating ROIs).

The present investigation focused on neural responding during the execution of cognitive tasks measuring impulsivity. It, therefore, does not warrant any conclusions on the relationship between brain activity and self-report measures of impulsivity, as questionnaire-derived trait assessments often demonstrate limited correlations with performance-based assessments of impulsivity (Sharma et al., 2014). Future work may investigate whether individual differences in trait impulsivity relate to the network identified here. In the present work, we focused on the two best-characterised dimensions of impulsivity that were also most commonly investigated with fMRI. Some models suggest sustained attention (the ability to keep one’s attention focused over time) and risk taking as additional components of impulsivity (Strickland & Johnson, 2021); however, there is substantial variance in proposed behavioural assessments. A meta-analysis of fMRI studies investigating sustained attention byLangner and Eickhoff (2013)has reported activations in regions largely overlapping with those identified here in the response inhibition network. Risky behaviours rarely play a substantial role in theoretical models of impulsivity and have been measured using the probability discounting task and Balloon Analog Risk Task (Lejuez et al., 2002). fMRI investigations during these tasks have revealed regions within the DCS network and parts of the multiple demand network (Miedl et al., 2012;Peters & Büchel, 2009;Schonberg et al., 2012;Seaman et al., 2018), suggesting overlapping activation with regions found in our meta-analyses. Therefore, the network described here may provide a largely comprehensive description of the neurocircuitry associated with the multi-dimensional construct of impulsivity.

## 5 Conclusions

Taken together, our findings reinforce insights from previous behavioural research and provide substantial evidence for the multi-dimensional nature of impulsivity on the neural level. In particular, we identified and characterised two non-overlapping neurocognitive systems linked to processes underlying impulsive and controlled decision-making and action control. Each of these was centred in a distinct large-scale network of brain organisation. The first was located in the default-mode network associated with value-based judgements and goal-directed decision-making, the second was distributed across higher-order networks related to executive functions of action selection, planning, and updating. These systems were found to be organised into four specialised communities of medial frontal, cingulo-insular, fronto-parietal, and temporal regions. Interactions between the communities and their coordination may affect the impulsivity of our behaviour and decision-making, with the modulation of community integration by serotonin emerging as a possible mechanism. Overall, our findings underscore the necessity for a comprehensive dimensional ontology encompassing symptoms, cognitive processes, and neural systems to effectively address impulsivity in a transdiagnostic manner (Berlin & Hollander, 2014). The research domain criteria framework of the NIH (Insel et al., 2010) has already taken steps in such a direction, with reward valuation and response selection/inhibition forming two separate components—but only the latter refers to impulsivity. Such developments, however, have yet to penetrate clinical research and practice.

## Supplementary Material

Supplementary Material

## Data Availability

All scripts and resources utilised in the analysis reported here can be accessed in a public repository on github athttps://github.com/MartinGell/Impulsivity_networks. All data used are freely available fromhttps://db.humanconnectome.org/app/template/Login.vmandhttp://fcon_1000.projects.nitrc.org/indi/enhanced/data.html.
