# Supplementary material for: Charting the brain networks of impulsivity: Meta-analytic synthesis, functional connectivity modelling, and neurotransmitter associations
Source: Imaging Neurosci (Camb). 2024 Sep 25;2:imag-2-00295. doi: 10.1162/imag_a_00295 (PMC12290857; doi:10.1162/imag_a_00295)
Supplement: Supplementary Material [file imag_a_00295-supp.pdf]

Supplementary Figures and Tables to Gell et al., 2022

1. Supplementary Figures

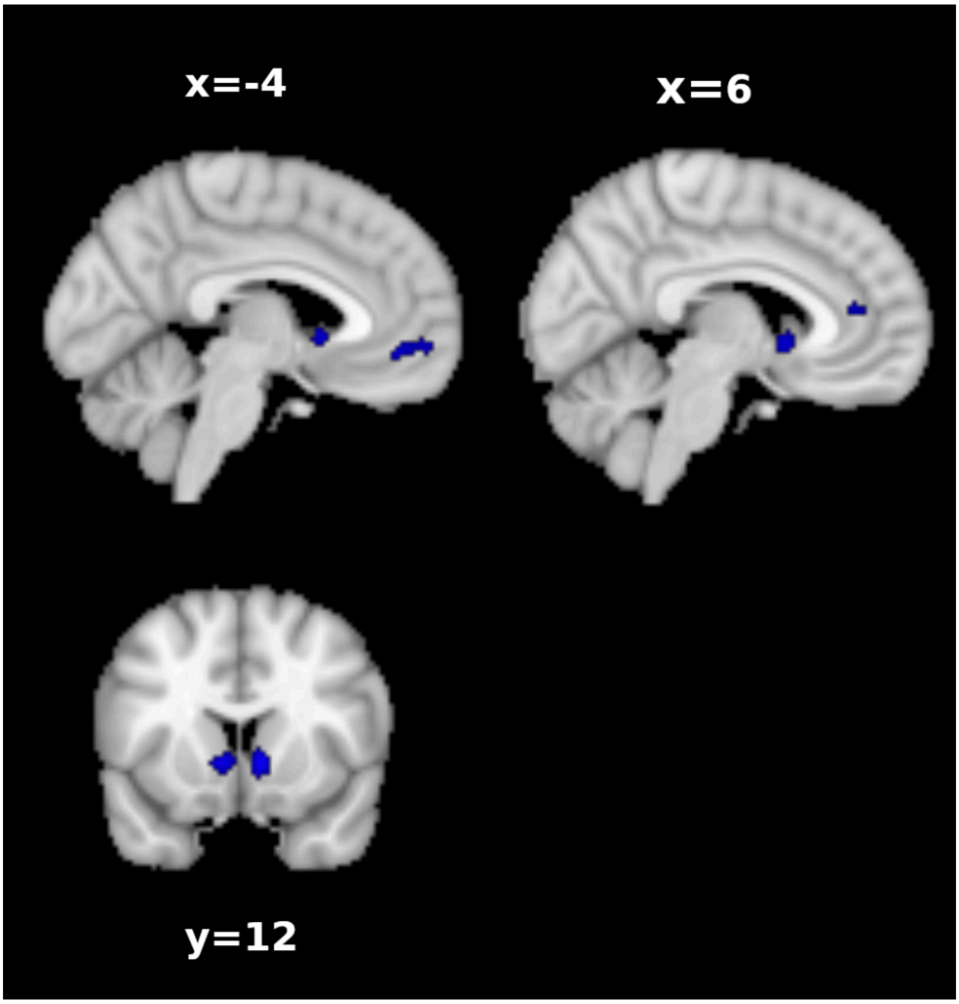

Supplementary Figure 1. Conjunction of impulsive responding and subjective value meta-analyses in DCS.

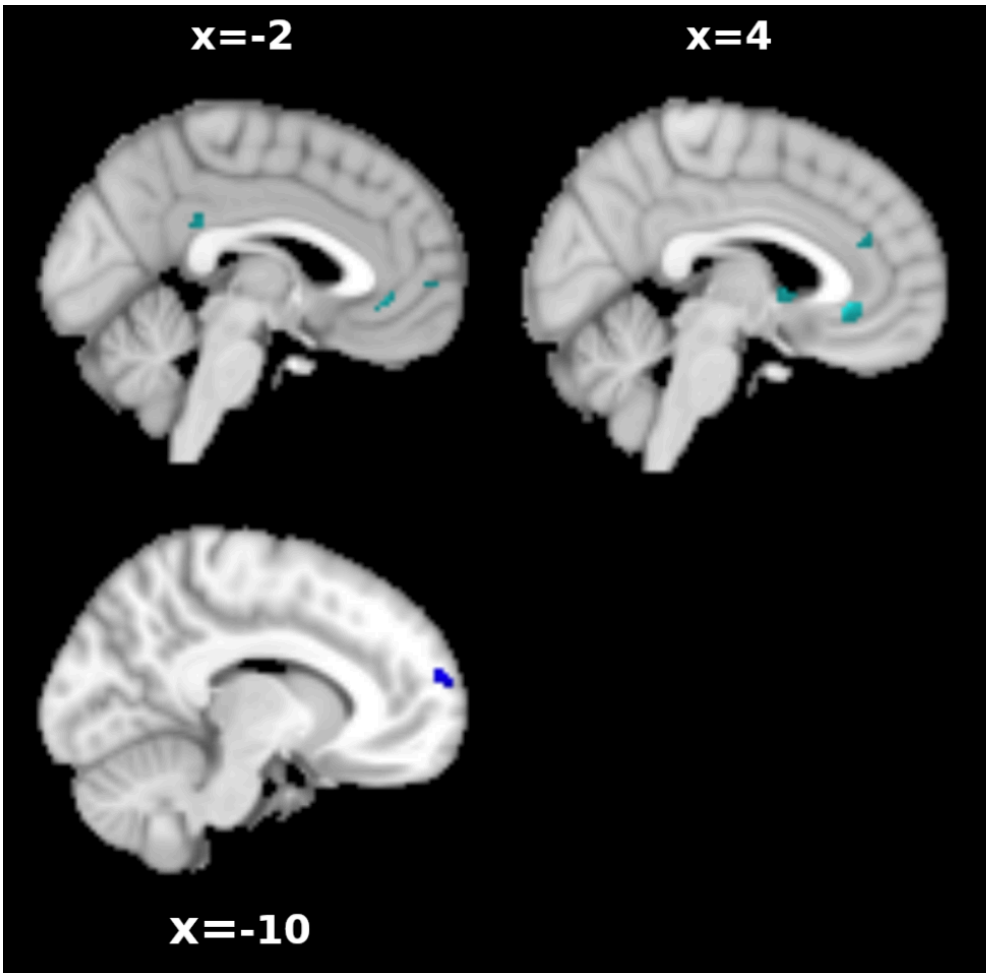

Supplementary Figure 2. Contrast analysis of impulsive responding and subjective value meta-analyses in DCS. Impulsive responding > subjective value in blue and opposite contrast in cyan.

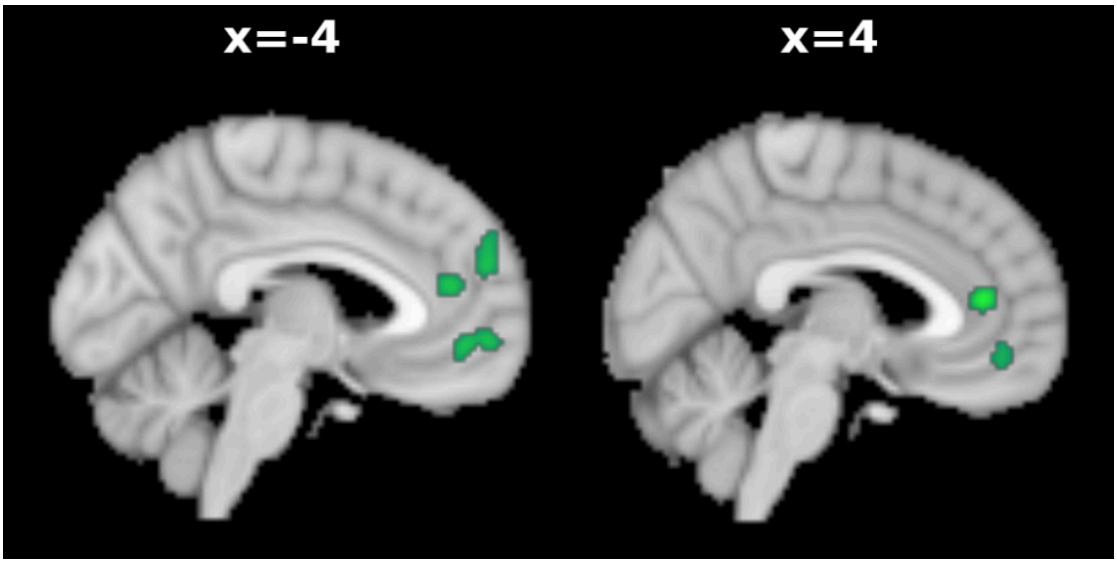

Supplementary Figure 3. Meta-analysis of impulsive responding in DCS without studies correlating activity with k.

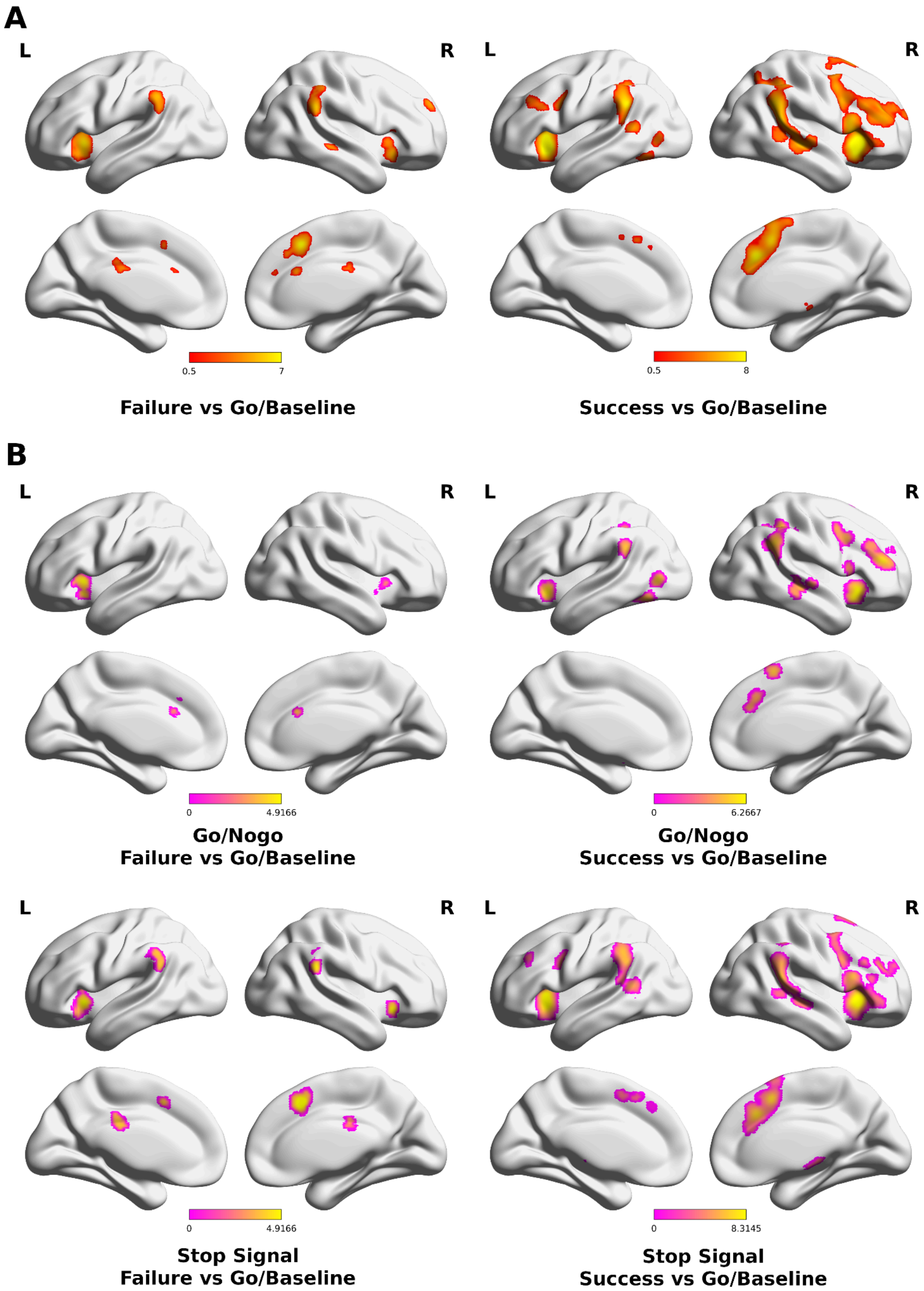

Supplementary Figure 4. Results of failure of inhibition against go/baseline and successful inhibition against go/baseline meta-analyses in (A) combined Stop Signal and Go/Nogo as well as (B) separately for each task.

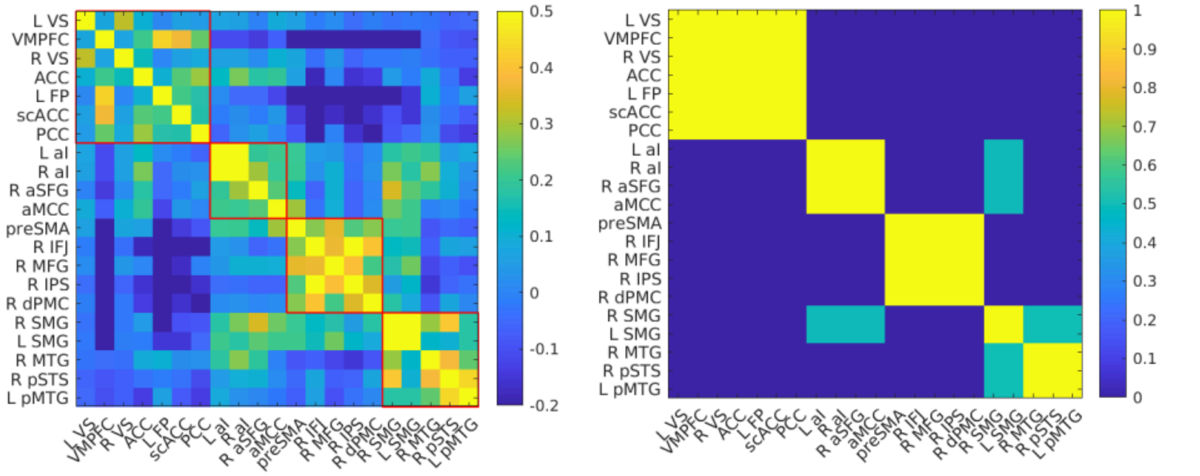

Supplementary Figure 6. Community detection results in the replication sample. Resulting communities on the left and agreement matrix on the right side (1 = all 1000 repeats yielded the same clustering solution).

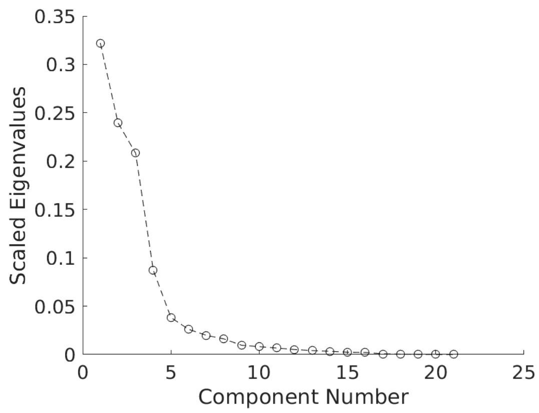

Supplementary Figure 7. Scree plot showing the eigenvalues of each component.

Supplementary Tables

Supplementary table 1: Checklist for Neuroimaging Meta-Analyses by Müller et al. (2018)

The research question was specifically defined	YES, and it included the following contrasts:<br><br>1) Impulsive choice (smaller sooner > larger later, immediate > delay, β > δ, correlation with k)<br>2) Controlled choice (larger later > smaller sooner, delay > immediate, δ > β, correlation with k)<br>3) Subjective value (parametric modulation of and correlation with subjective value)<br>4) Impulsive action (commission error > go/baseline/rest)<br>5) Controlled action (successful inhibition > go/baseline/rest)<br><br>The specific contrasts are reports in the method section
The literature search was systematic	YES, it included the following keywords in the following databases:<br><br>1. Delay consequence sensitivity<br>“delay discounting” or “temporal discounting” or “delayed reward” and “fMRI” or “functional magnetic resonance imaging”<br><br>2. Response inhibition<br>“stop signal task” or “go nogo task” or “response inhibition” or “inhibition” or "action withholding" or "action cancellation" or "action inhibition" or "motor inhibition" or "inhibitory control" and “fMRI” or “functional magnetic resonance imaging”<br><br>Databases: PubMed, Web of Science
Detailed inclusion and exclusion criteria were applied	YES, and reasons of non-standard criteria were:<br><br>Inclusion of: fMRI studies, healthy participants, mean age >= 12, contrast of interest, no region of interest, no pharmacological interventions or connectivity-based analyses
Sample overlap was taken into account	YES, using the following method:<br><br>In cases of partly overlapping subject groups (e.g. as in the case of Kable 2007 and Kable 2010), coordinates were pooled to form a single experiment and the smaller N of the two original experiments was used as the input to ALE.
All experiments used the same search coverage (state how brain coverage was assessed and how small volume corrections and conjunctions were taken into account)	YES, the search coverage was the following:<br><br>- whole-brain coverage only<br>- exclusion of ROI studies

Studies are converted to a common reference space	YES, using the following conversion(s):<br><br>Coordinates reported in Talairach space were converted to MNI space (Lancaster et al., 2007)
Data extraction was conducted by two investigators (ideal case) or double-checked by the same investigator (state how double-checking was performed)	YES, the following authors:<br><br>- MG, VM, EC checked inclusion criteria<br>- MG extracted coordinates<br>- MG and VK extracted other info: age, sex, sample size, contrast, space<br>- VK and VM double-checked the following data: inclusion criteria, extracted coordinates, all other infos
The paper includes a table with at least the references, basic study description (e.g., for fMRI tasks, stimuli), contrasts and basic sample descriptions (e.g., size, mean age and gender distribution, specific characteristics) of the included studies, source of information (e.g., contact with authors), reference space	YES, and also the following data:<br><br>- If further information was received by the authors
The study protocol and all analyses was planned before- hand, including the methods and parameters used for inference, correction for multiple testing, etc.	YES. The meta-analysis was not pre-registered; however, all analyses including methods and recommended parameters used for inference were planned before starting the literature search
The paper includes meta-analytic diagnostics	No diagnostics are reported given that all analyses included at least 21 experiments and a cFWE correction. As shown in Eickhoff 2016 results are robust with regard to being driven by only a few experiments.

Supplementary Methods to Gell et al., 2022

1. Main Effect Thresholding and Masking

In order to account for the higher power of the controlled action > baseline/go meta-analysis (i.e. large discrepancy in the number of included studies: 26 in impulsive action vs 103 in controlled action) we used a subsampling procedure. This was achieved by iteratively sub-sampling 26 experiments from the full controlled action database 10 000 times. In each iteration a meta-analysis was calculated and for each voxel an absence or presence of a significant main effect was recorded. Thus a frequency of significant results across the 10 000 iterations for each voxel was obtained. The resulting probabilistic map represents the number of times each voxel participated in a main effect over the permutation procedure as a proportion of the total number of permutations. Finally, this probabilistic map was thresholded at the 90th percentile to remove voxels with a very low probability of participating in a main effect and used to mask the controlled choice main effect map before computing the contrast (see figure x in supplement for illustration of this procedure). An illustration of this procedure is displayed below.

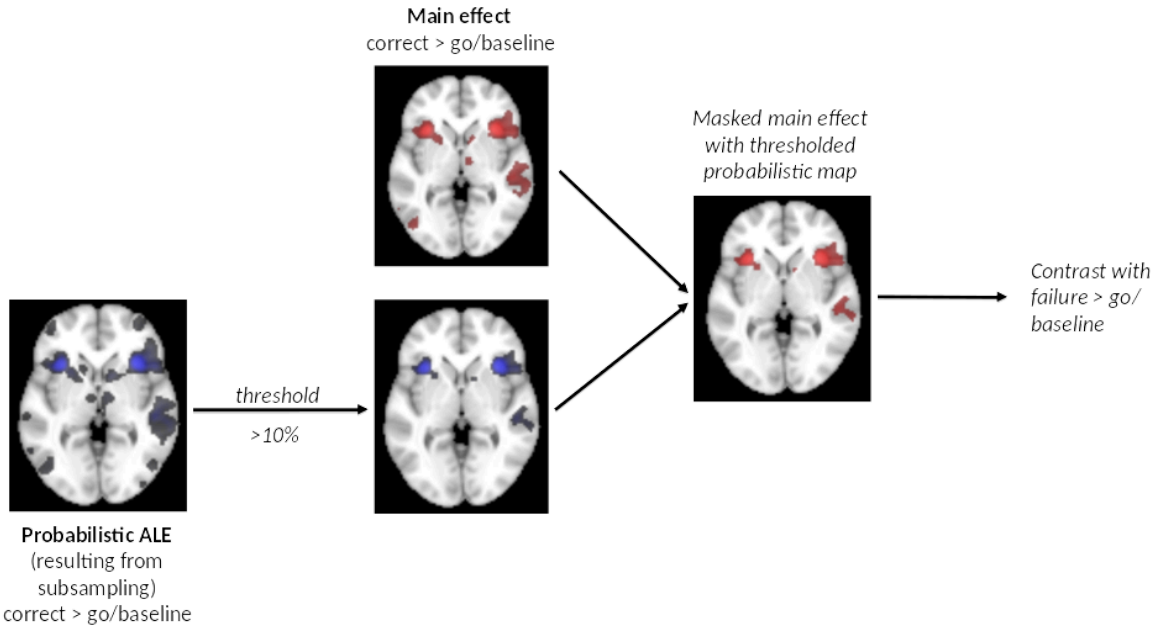

Figure S1. Main effect masking procedure

2. fMRIPrep preprocessing

Anatomical data preprocessing

T1-weighted (T1w) images were corrected for intensity non-uniformity (INU) with `N4BiasFieldCorrection` (Tustison et al., 2010), distributed with ANTs 2.2.0 (Avants, Epstein,

Grossman, and Gee, 2008). The T1w-reference was then skull-stripped with a *Nipype* implementation of the `antsBrainExtraction.sh` workflow (from ANTs), using OASIS30ANTs as target template. Brain tissue segmentation of cerebrospinal fluid (CSF), white-matter (WM) and gray-matter (GM) was performed on the brain-extracted T1w using `fast` [FSL 5.0.9, Zhang, Brady, and Smith, 2001)].

A T1w-reference map was computed after registration of 2 T1w images (after INU-correction) using `mri_robust_template` [FreeSurfer 6.0.1, Reuter, Rosas, and Fischl, 2010)]. Brain surfaces were reconstructed using `recon-all` [FreeSurfer 6.0.1, Dale, Fischl, and Sereno, 1999)], and the brain mask estimated previously was refined with a custom variation of the method to reconcile ANTs-derived and FreeSurfer-derived segmentations of the cortical gray-matter of Mindboggle (Klein et al., 2017). Volume-based spatial normalization to two standard spaces (MNI152NLin6Asym, MNI152NLin2009cAsym) was performed through nonlinear registration with `antsRegistration` (ANTs 2.2.0), using brain-extracted versions of both T1w reference and the T1w template. The following templates were selected for spatial normalization: *FSL's MNI ICBM 152 non-linear 6th Generation Asymmetric Average Brain Stereotaxic Registration Model* [Evans, Janke, Collins, and Baillet, 2012), RRID:SCR_002823; TemplateFlow ID: MNI152NLin6Asym], *ICBM 152 Nonlinear Asymmetrical template version 2009c* [Fonov, Evans, McKinstry, Almli and Collins, et al., 2009), RRID:SCR_008796; TemplateFlow ID: MNI152NLin2009cAsym].

Functional data preprocessing

For the BOLD runs found per subject (across all tasks and sessions), the following preprocessing was performed. First, a reference volume and its skull-stripped version were generated using a custom methodology of *fMRIPrep*. Head-motion parameters with respect to the BOLD reference (transformation matrices, and six corresponding rotation and translation parameters) are estimated before any spatiotemporal filtering using `mcflirt` [FSL 5.0.9, (Jenkinson, Bannister, Brady, and Smith, 2002)]. BOLD runs were slice-time corrected using `3dTshift` from AFNI 20160207 (Cox and Hyde, 1997). Susceptibility distortion correction (SDC) was omitted. The BOLD reference was then co-registered to the T1w reference using `bbregister` (FreeSurfer) which implements boundary-based registration (Greve and Fischl, 2009). Co-registration was configured with six degrees of freedom. The BOLD time-series were resampled onto the following surfaces (FreeSurfer reconstruction nomenclature): *fsaverage*.

The BOLD time-series (including slice-timing correction when applied) were resampled onto their original, native space by applying the transforms to correct for head-motion. These resampled BOLD time-series will be referred to as *preprocessed BOLD in original space*, or just *preprocessed BOLD*. The BOLD time-series were resampled into standard space, generating a *preprocessed BOLD run in MNI152NLin6Asym space*. First, a reference volume and its skull-stripped version were generated using a custom methodology of *fMRIPrep*. *Grayordinates* files (Glasser et al., 2013) containing 91k samples were also generated using the highest-resolution ``fsaverage`` as intermediate standardized surface space.

Automatic removal of motion artifacts using independent component analysis [ICA-AROMA, Pruim et al., (2015)] was performed on the *preprocessed BOLD on MNI space* time-series after removal of non-steady state volumes and spatial smoothing with an isotropic, Gaussian kernel of 6mm FWHM (full-width half-maximum). Corresponding "non-aggresively" denoised runs were produced after such Smoothing. Additionally, the "aggressive" noise-regressors were collected and placed in the corresponding confounds file. Several confounding time-series were calculated based on the *preprocessed BOLD*: framewise displacement (FD), DVARS and three region-wise global signals. FD was computed using two formulations following Power (absolute sum of relative motions, Power et al., (2014)) and Jenkinson (relative root mean square displacement between affines, Jenkinson et al. (2002)). FD and DVARS are calculated for each functional run, both using their implementations in *Nipype* [following the definitions by Power et al., (2014)]. The three global signals are extracted within the CSF, the WM, and the whole-brain masks.
Additionally, a set of physiological regressors were extracted to allow for component-based noise correction [*CompCor*, (Behzadi, Restom, Liau and Liu, 2007)]. Principal components are estimated after high-pass filtering the *preprocessed BOLD* time-series (using a discrete cosine filter with 128s cut-off) for the two *CompCor* variants: temporal (tCompCor) and anatomical (aCompCor). tCompCor components are then calculated from the top 5% variable voxels within a mask covering the subcortical regions. This subcortical mask is obtained by heavily eroding the brain mask, which ensures it does not include cortical GM regions. For aCompCor, components are calculated within the intersection of the aforementioned mask and the union of CSF and WM masks calculated in T1w space, after their projection to the native space of each functional run (using the inverse BOLD-to-T1w transformation). Components are also calculated separately within the WM and CSF masks. For each CompCor decomposition, the *k* components with the largest singular values are retained, such that the retained components' time series are sufficient to explain 50 percent of variance across the nuisance mask (CSF, WM, combined, or temporal). The remaining components are dropped from consideration.

The head-motion estimates calculated in the correction step were also placed within the corresponding confounds file. The confound time series derived from head motion estimates and global signals were expanded with the inclusion of temporal derivatives and quadratic terms for each (Satterthwaite et al., 2013). Frames that exceeded a threshold of 0.5 mm FD or 1.5 standardised DVARS were annotated as motion outliers. All resamplings can be performed with *a single interpolation step* by composing all the pertinent transformations (i.e. head-motion transform matrices, susceptibility distortion correction when available, and co-registrations to anatomical and output spaces). Gridded (volumetric) resamplings were performed using `antsApplyTransforms` (ANTs), configured with Lanczos interpolation to minimize the smoothing effects of other kernels (Lanczos, 1964). Non-gridded (surface) resamplings were performed using `mri_vol2surf` (FreeSurfer).

Many internal operations of *fMRIPrep* use *Nilearn* 0.6.2 (Abraham et al., 2014), mostly within the functional processing workflow. For more details of the pipeline, see [the section corresponding to workflows in *fMRIPrep*'s documentation](https://fmriprep.readthedocs.io/en/latest/workflows.html "FMRIPrep's documentation").

Copyright Waiver

The above boilerplate text was automatically generated by fMRIPrep with the express intention that users should copy and paste this text into their manuscripts *unchanged*. It is released under the [CC0](https://creativecommons.org/publicdomain/zero/1.0/) license.

References

Esteban, O., Markiewicz, C., Blair, R. W., Moodie, C., Isik, A. I., Erramuzpe Aliaga, A., Kent, J., Goncalves, M., DuPre, E., Snyder, M., Oya, H., Ghosh, S., Wright, J., Durnez, J., Poldrack, R., & Gorgolewski, K. J. (2019). fMRIPrep: A robust preprocessing pipeline for functional MRI. *Nature Methods, 16*, 111-116. [DOI: 10.1038/s41592-018-0235-4]

Esteban, O., Blair, R., Markiewicz, C. J., Berleant, S. L., Moodie, C., Ma, F., Isik, A. I., Erramuzpe, A., Kent, J. D., Goncalves, M., DuPre, E., Sitek, K. R., Gomez, D. E. P., Lurie, D. J., Ye, Z., Poldrack, R. A., & Gorgolewski, K. J. (2018). fMRIPrep 20.1.1 . *Software*. [DOI: 10.5281/zenodo.852659]

Ciric, R., Thompson, W. H., Lorenz, R., Goncalves, M., MacNicol, E., Markiewicz, C. J., Halchenko, Y. O., Ghosh, S. S., Gorgolewski, K. J., Poldrack, R. A., & Esteban, O. (2022). TemplateFlow: FAIR-sharing of multi-scale, multi-species brain models. *Nature Methods, 19*, 1568-1571. [DOI: 10.1038/s41592-022-01681-2]

Gorgolewski, K., Burns, C. D., Madison, C., Clark, D., Halchenko, Y. O., Waskom, M. L., & Ghosh, S. (2011). Nipype: A flexible, lightweight and extensible neuroimaging data processing framework in Python. *Frontiers in Neuroinformatics, 5*, 13. [DOI: 10.3389/fninf.2011.00013]

Gorgolewski, K. J., Esteban, O., Markiewicz, C. J., Ziegler, E., Ellis, D. G., Notter, M. P., Jarecka, D., Johnson, H., Burns, C., Manhães-Savio, A., Hamalainen, C., Yvernault, B., Salo, T., Jordan, K., Goncalves, M., Waskom, M., Clark, D., Wong, J., Loney, F., ... Ghosh, S. (2018). Nipype. *Software*. [DOI: 10.5281/zenodo.596855]

Tustison, N. J., Avants, B. B., Cook, P. A., Zheng, Y., Egan, A., Yushkevich, P. A., & Gee, J. C. (2010). N4ITK: Improved N3 Bias Correction. *IEEE Transactions on Medical Imaging, 29*(6), 1310-1320. [DOI: 10.1109/TMI.2010.2046908]

Dale, A. M., Fischl, B., & Sereno, M. I. (1999). Cortical Surface-Based Analysis: I. Segmentation and Surface Reconstruction. *NeuroImage, 9*(2), 179-194. [DOI: 10.1006/nimg.1998.0395]

Klein, A., Ghosh, S. S., Bao, F. S., Giard, J., Häme, Y., Stavsky, E., Lee, N., Rossa, B., Reuter, M., Neto, E. C., & Keshavan, A. (2017). Mindboggling morphometry of human brains. *PLOS Computational Biology, 13*(2), e1005350. [DOI: 10.1371/journal.pcbi.1005350]

Mazziotta, J. C., Toga, A. W., Evans, A., Fox, P., & Lancaster, J. (1995). A Probabilistic Atlas of the Human Brain: Theory and Rationale for Its Development: The International

Consortium for Brain Mapping (ICBM). *NeuroImage*, *2*(2, Part A), 89-101. doi:10.1006/nimg.1995.1012

Pruim, R. H. R., Mennes, M., Buitelaar, J. K., & Beckmann, C. F. (2015). Evaluation of ICA-AROMA and alternative strategies for motion artifact removal in resting state fMRI. NeuroImage, 112, 278–287. doi: 10.1016/j.neuroimage.2015.02.063

Fonov, V. S., Evans, A. C., McKinstry, R. C., Almli, C. R., & Collins, D. L. (2009). Unbiased nonlinear average age-appropriate brain templates from birth to adulthood. *NeuroImage*, *47*(Supplement 1), S102. doi:10.1016/S1053-8119(09)70884-5

Evans, A. C., Janke, A. L., Collins, D. L., & Baillet, S. (2012). Brain templates and atlases. *NeuroImage*, *62*(2), 911-922. doi:10.1016/j.neuroimage.2012.01.024

Avants, B. B., Epstein, C. L., Grossman, M., & Gee, J. C. (2008). Symmetric diffeomorphic image registration with cross-correlation: Evaluating automated labeling of elderly and neurodegenerative brain. *Medical Image Analysis*, *12*(1), 26-41. doi:10.1016/j.media.2007.06.004

Zhang, Y., Brady, M., & Smith, S. (2001). Segmentation of brain MR images through a hidden Markov random field model and the expectation-maximization algorithm. *IEEE Transactions on Medical Imaging*, *20*(1), 45-57. doi:10.1109/42.906424

Wang, S., Peterson, D. J., Gatenby, J. C., Li, W., Grabowski, T. J., & Madhyastha, T. M. (2017). Evaluation of Field Map and Nonlinear Registration Methods for Correction of Susceptibility Artifacts in Diffusion MRI. *Frontiers in Neuroinformatics*, *11*, Article 17. doi:10.3389/fninf.2017.00017

Huntenburg, J. M. (2014). Evaluating nonlinear coregistration of BOLD EPI and T1w images (Master's thesis). Freie Universität. Retrieved from http://hdl.handle.net/11858/00-001M-0000-002B-1CB5-A

Treiber, J. M., White, N. S., Steed, T. C., Bartsch, H., Holland, D., Farid, N., McDonald, C. R., Carter, B. S., Dale, A. M., & Chen, C. C. (2016). Characterization and Correction of Geometric Distortions in 814 Diffusion Weighted Images. *PLOS ONE*, *11*(3), e0152472. doi:10.1371/journal.pone.0152472

Jenkinson, M., & Smith, S. (2001). A global optimisation method for robust affine registration of brain images. *Medical Image Analysis*, *5*(2), 143-156. doi:10.1016/S1361-8415(01)00036-6

Jenkinson, M., Bannister, P., Brady, M., & Smith, S. (2002). Improved Optimization for the Robust and Accurate Linear Registration and Motion Correction of Brain Images. *NeuroImage, 17*(2), 825-841. doi:10.1006/nimg.2002.1132.

Greve, D. N., & Fischl, B. (2009). Accurate and robust brain image alignment using boundary-based registration. *NeuroImage, 48*(1), 63-72. doi:10.1016/j.neuroimage.2009.06.060.

Power, J. D., Mitra, A., Laumann, T. O., Snyder, A. Z., Schlaggar, B. L., & Petersen, S. E. (2014). Methods to detect, characterize, and remove motion artifact in resting state fMRI. *NeuroImage, 84*, 320-341. doi:10.1016/j.neuroimage.2013.08.048.

Satterthwaite, T. D., Elliott, M. A., Gerraty, R. T., Ruparel, K., Loughead, J., Calkins, M. E., ... Wolf, D. H. (2013). An improved framework for confound regression and filtering for control of motion artifact in the preprocessing of resting-state functional connectivity data. *NeuroImage, 64*(1), 240-256. doi:10.1016/j.neuroimage.2012.08.052.

Abraham, A., Pedregosa, F., Eickenberg, M., Gervais, P., Mueller, A., Kossaifi, J., ... Varoquaux, G. (2014). Machine learning for neuroimaging with scikit-learn. *Frontiers in Neuroinformatics, 8*. doi:10.3389/fninf.2014.00014.

Lanczos, C. (1964). Evaluation of Noisy Data. *Journal of the Society for Industrial and Applied Mathematics Series B Numerical Analysis, 1*(1), 76-85. doi:10.1137/0701007.

Behzadi, Y., Restom, K., Liau, J., & Liu, T. T. (2007). A component based noise correction method (CompCor) for BOLD and perfusion based fMRI. *NeuroImage, 37*(1), 90-101. doi:10.1016/j.neuroimage.2007.04.042.

Glasser, M. F., Sotiropoulos, S. N., Wilson, J. A., Coalson, T. S., Fischl, B., Andersson, J. L., ... Jenkinson, M. (2013). The minimal preprocessing

Reuter, M., Rosas, H. D., & Fischl, B. (2010). Highly accurate inverse consistent registration: A robust approach. *NeuroImage*, 53(4), 1181-1196. doi:10.1016/j.neuroimage.2010.07.020

Cox, R. W., & Hyde, J. S. (1997). Software tools for analysis and visualization of fMRI data. *NMR in Biomedicine*, 10(4-5), 171-178. doi:10.1002/(SICI)1099-1492(199706/08)10:4/5<171::AID-NBM453>3.0.CO;2-L

Posse, S., Wiese, S., Gembris, D., Mathiak, K., Kessler, C., Grosse-Ruyken, M.-L., Elghahwagi, B., Richards, T., Dager, S. R., & Kiselev, V. G. (1999). Enhancement of BOLD-contrast sensitivity by single-shot multi-echo functional MR imaging. *Magnetic Resonance in Medicine*, 42(1), 87-97. doi:10.1002/(SICI)1522-2594(199907)42:1<87::AID-MRM13>3.0.CO;2-O

Andersson, J. L. R., Skare, S., & Ashburner, J. (2003). How to correct susceptibility distortions in spin-echo echo-planar images: application to diffusion tensor imaging. *NeuroImage*, 20(2), 870-888. doi:10.1016/S1053-8119(03)00336-7

Patriat, R., Reynolds, R. C., & Birn, R. M. (2017). An improved model of motion-related signal changes in fMRI. *NeuroImage*, 144(Part A), 74-82. doi:10.1016/j.neuroimage.2016.08.051

Included Studies for Delay Consequence Sensitivity

Impulsive Decision-making					
Author	N	Contrast	Space	Were in-scanner presented rewards adapted based on subjective value?	Source (of coordinates)
Albrecht et al., 2011	28	immediate > delay	TAL	not subject adapted	Table S4
Albrecht et al., 2013	30	now > later	TAL	not subject adapted	Table S4
Banich et al., 2013	29	now > later	MNI	not subject adapted	Table 2
Christakou et al., 2011	40	immediate > delay	TAL	subject adapted	Table 1
de Water et al., 2017	58	correlation of sensitivity to immediate reward in immediate > delay	MNI	not subject adapted	Table S5
Deshpande et al., 2019	54	immediate and delay > delay and delay	MNI	subject adapted	Table S
Elton et al., 2017	95	positive correlation with impulsive choice ration (similar to k) in immediate & delay > control	MNI	not subject adapted	Table 1
Eppinger et al., 2012	30	beta (immediate & delay > all choice options)	TAL	not subject adapted	In text, figure 2
Faralla et al., 2015	28	(immediate > delay) > control	MNI	not subject adapted	Table 5
Hamilton et al. 2020	27	SS > LL	MNI	subject adapted	neurovault
Kable and Glimcher 2010	22	immediate and delay > delay and delay	MNI	subject adapted	Table 1
Luo et al., 2009	37	immediate > delay	MNI	subject adapted	Table 2

Mavrogiorgou et al., 2016	20	immediate > delay	MNI	subject adapted	Table 2
McClure et al., 2004	14	beta (immediate & 0.5*delay)	MNI	not subject adapted	Table S1
Norman et al., 2017	20	immediate > delay	TAL	subject adapted	Table S1
Pine et al., 2009	24	correlation with discount factor D	MNI	not subject adapted	Table S5
Samanez-Larkin et al., 2011	25	beta	TAL	not subject adapted	Table 1
Sripada et al., 2011	20	immediate and delay > delay and delay	MNI	not subject adapted	Table 1
Wittmann et al., 2010	13	immediate > delay	TAL	not subject adapted	table S7
Xu et al., 2009	18	immediate and delay > delay and delay	MNI	not subject adapted	table S2
Zhuang et al., 2020	16	sooner > delayed (main effect of delay decision)	MNI	not subject adapted	Table 2

Controlled Decision-making					
Author	N	Contrast	Space	Were in-scanner presented rewards adapted based on subjective value?	Source (of coordinates)
Banich et al., 2013	29	later > now	MNI	not subject adapted	Table 2
Christakou et al., 2011	40	delay > immediate	TAL	subject adapted	Table 1
de Water et al., 2017	58	delay > immediate	MNI	not subject adapted	Table S4
	58	impatience during delay > immediate	MNI	not subject adapted	Table S5
Deshpande et al., 2019	54	delay and delay > immediate and delay	MNI	subject adapted	Table S
Elton et al., 2017	95	neg corr. with impulsive choice ratio	MNI	not subject adapted	Table 1
Eppinger et al., 2012	30	delta (delay & immediate)	TAL	not subject adapted	In text, Figure 2
Faralla et al., 2015	28	(delay > immediate) > control	MNI	not subject adapted	Table 6
Hamilton et al. 2020	27	LL > SS	MNI	subject adapted	neurovault
Hill et al., 2017	25	delay > immediate	MNI	subject adapted	Table S2
Kable and Glimcher 2007	10	delay > immediate	TAL	subject adapted	Table S5
Kable and Glimcher 2010	22	delay and delay > immediate and delay	TAL	subject adapted	Table 1: reverse contrast
King et al., 2016	36	LL > SS	MNI	subject adapted	authors
Laube et al., 2020	48	LL > SS	MNI	subject adapted	In text, Figure 3

Luo et al., 2009	37	delay > immediate	MNI	subject adapted	Table 2
Luo et al., 2012	21	LL > SS	MNI	subject adapted	Table 1
McClure et al., 2004	14	delta (delay & immediate > baseline)	MNI	not subject adapted	Table S2
Miedl et al., 2015	15	delay > immediate	TAL	not subject adapted	Table 4
Norman et al., 2017	20	delay > immediate	TAL	subject adapted	Table S1
O'Connell et al., 2018	26	delay > immediate	MNI	subject adapted	Figure S3
Samanez-Larkin et al., 2011	25	delta	TAL	not subject adapted	Table 1
Sripada et al., 2011	20	delay > immediate	MNI	not subject adapted	Table 1
Van den Bos et al., 2014	22	LL > SS	MNI	subject adapted	Table 2
Waegeman et al., 2014	41	delay > immediate	MNI	not subject adapted	Table 2
Wang et al., 2017	21	delay > immediate	MNI	not subject adapted	Table 3
Wittmann et al., 2007	13	delay > immediate	TAL	not subject adapted	Table 1
Wittmann et al., 2010	13	delay > immediate	TAL	not subject adapted	Table S7
Zhuang et al., 2020	16	delayed > sooner (main effect of delay decision)	MNI	not subject adapted	Table 2

Subjective value					
Author	N	Contrast	Space	Were in-scanner presented rewards adapted based on subjective value?	Source (of coordinates)
Castrellon et al., 2019	21	subjective value (parametric modulation)	MNI	not subject adapted	Table 2
Cox and Kable 2014	20	subjective value (parametric modulation)	MNI	subject adapted	Table 1
Eppinger et al., 2017	50	correlation with subjective value	TAL	subject adapted	In text, Figure 5
Hare et al., 2014	25	correlation with discounted stimulus value (SV)	MNI	not subject adapted	Table 3
Jimura et al., 2013	43	correlation with subjective value	TAL	subject adapted	Table 1
Kable and Glimcher 2007	10	correlation with subjective value, discount rate	TAL	subject adapted	Table S5
Kable and Glimcher 2010	22	correlation with subjective value	TAL	subject adapted	Table 1
King et al., 2016	36	subjective value (parametric modulation)	MNI	subject adapted	authors
Lempert et al., 2017	35	subjective value (parametric modulation)	MNI	not subject adapted	Table 2
Liu et al., 2012	19	correlation with subjective value	TAL	subject adapted	Table 1
Luo et al., 2012	21	subjective value, stochasticity, discounting factor (k)	MNI	subject adapted	Table S2
Massar et al., 2015	23	correlation with subjective value	TAL	subject adapted	Table 1
Murawski, et al., 2012	13	subjective value > baseline	MNI	subject adapted	Table S5

O'Connell et al., 2018	26	subjective value (parametric modulation)	MNI	subject adapted	Figure S3
Peters and Büchel 2009	22	correlation with subjective value	MNI	subject adapted	Table S2
Peters and Büchel 2010	30	correlation with subjective value	MNI	subject adapted	In text
Pine et al., 2009	24	correlation with subjective value (discounted utility)	MNI	not subject adapted	Table S6
Prevost et al., 2010	18	correlation with subjective value	MNI	subject adapted (force)	Table 1
Ripke et al., 2012	27	subjective value (parametric modulation)	MNI	subject adapted	Tables S1 and S3
Samanez-Larkin et al., 2011	25	correlation with subjective value	TAL	not subject adapted	Table S2
Sasse et al., 2017	22	subjective value (parametric modulation)	MNI	subject adapted	Table 3
Seaman et al., 2018	75	correlation with subjective value	MNI	not subject adapted	Table 3
Sripada et al., 2011	20	correlation with subjective value	MNI	not subject adapted	Table 1
Wang et al., 2014	28	subjective relative value, corr. with value of immediate option	MNI	not subject adapted (adapted from pilot study matching in age and gender)	Table 1
Wiehler et al., 2017	23	subjective value (parametric modulation)	MNI	subject adapted	authors

Included Studies for Response Inhibition

Successful Inhibition Database					
Author	n	contrast	space	paradigm	type of error
Aron and Poldrack, 2006	13	Correct Stop > Correct Go	MNI	stop signal task	SM Table 2
Berkmann et al., 2014	60	Correct Stop > Correct Go	MNI	stop signal task	Table 2
Bobb et al., 2011	13	Correct Stop > Correct Go	MNI	stop signal task	Table 2
Boecker et al., 2011	15	Correct Stop > Correct Go	MNI*	stop signal task	SM Table 1
Boehler et al., 2010	15	Correct Stop > Go	MNI	stop signal task	Table 3
Cai and Leung, 2009	12	Correct Stop > Correct Go	MNI	stop signal task	Table 1
Cai and Leung, 2011	23	Correct Stop > Go	MNI	stop signal task	SM Table 1
Cai et al. 2014	19	Correct Stop > Correct Go	MNI	stop signal task	Table 2
Chen et al., 2015	25	Correct NoGo > Go	MNI	go/no-go	Table 2
Chevrier et al., 2007	14	correct stop > go	TAL	stop signal task	Table 1
Chikara et al., 2018	20	Correct Stop > Correct Go	MNI	stop signal task	Table 3A
Chikazoe et al., 2009a	22	Correct Stop > Correct uncertain go trials	MNI	stop signal task	Table 2
Chikazoe et al., 2009b	25	correct No-Go > infrequent correct Go	MNI	go/no-go	Table 1
				go/no-go	Table 2

Congdon et al., 2014	62	Correct Stop > Correct Go	MNI	stop signal task	Table 2
Coxon et al., 2016_1	20	Correct Stop > Correct Go	MNI	stop signal task	Table 2 (young)
Coxon et al., 2016_2	20	Correct Stop > Correct Go	MNI	stop signal task	Table 2 (old)
Czapla et al., 2017	21	Correct NoGo > Correct Go	MNI	go/no-go	SM Table 4
				go/no-go	SM Table 5
Dambacher et al., 2015	15	Correct NoGo > Go	TAL	go/no-go	Table 1
Fassbender et al., 2004	18	Correct Inhibitions > tonic activation	TAL	go/no-go	Table 2
Fauth-Bühler et al., 2012	18	successful stop > baseline	MNI	stop signal task	from authors
Fedota et al., 2015	16	Correct NoGo > Correct Go	MNI	go/no-go	Table 1
Fuentes-Claramonte et al., 2016	57	Correct NoGo > Correct frequent Go	MNI	go/no-go	Table 2
		Correct NoGo > Correct Infrequent Go		go/no-go	Table 2
Ganos et al., 2014	15	Correct Stop > Go	MNI	stop signal task	SM Table 2
Garavan et al., 1999	14	correct Inhibition > active baseline	TAL	go/no-go	Table 1
Garavan et al., 2002	14	correct Inhibition > active baseline	TAL	go/no-go	Table 1
Garavan et al., 2003	16	correct Inhibition > active baseline	TAL	go/no-go	Table 1
Geng et al., 2009	16	Correct NoGo > Correct Go	MNI	go/no-go	Table 3
Ghahremani et al., 2012	18	Correct Stop > Correct Go	MNI	stop signal task	Table 3

Harle et al., 2016	34	Correct Stop > Correct Go	TAL	stop signal task	SM Table 2
Hester et al., 2004a	15	successful inhibition vs. Baseline	TAL	go/no-go	Table 1
Hester et al., 2004b	15	Successful inhibition vs go	TAL	go/no-go	Table 3
Hough et al., 2016	22	Correct NoGo > Correct Go	MNI	go/no-go	SM Table
Hsu et al., 2017	20	Correct NoGo > Go	MNI	go/no-go	Table 2
Hughes et al., 2012	10	Correct stop > baseline	MNI	stop signal task	Table 4
Hughes et al., 2013	15	Correct stop > baseline	MNI	stop signal task	Table 3
Jahfari et al., 2011	20	Correct Stop > Go	MNI	stop signal task	Table 4
Jahfari et al., 2012	16	Correct Stop > Go	MNI	stop signal task	Table 5
Jahfari et al., 2015	23	Correct Stop > Go	MNI	stop signal task	Table 3
Kaladjian et al., 2007	21	correct No-Go > Correct Go	TAL	go/no-go	Table 3
Kaladjian et al., 2009a	20	correct No-Go > Correct Go	TAL	go/no-go	Table 3
Kaladjian et al., 2009b	10	correct No-Go > Correct Go	TAL	go/no-go	Table 3
		correct No-Go > Correct Go		go/no-go	Table 3
Kelly et al., 2004	15	Successful inhibition vs baseline	TAL	go/no-go	Table1
Kenner et al., 2010	24	Correct Stop > Correct Go	MNI	stop signal task	SM Table 3
Kiehl et al., 2000	14	correct Inhibition > active baseline	MNI	go/no-go	Table 1
Ko et al., 2014	23	Correct NoGo > Go	MNI	go/no-go	Table 2

Ko et al., 2016	32	Correct Stop > Correct Go	MNI	stop signal task	Table 1A
		Correct Stop > Correct Go		stop signal task	Table 1B
Köhler et al., 2018	33	Correct NoGo > Correct Go	MNI	go/no-go	SM Table 1
Lavallee et al., 2014	21	Correct Stop > Go	MNI	stop signal task	Table 2
Liddle et al. , 2001	16	correctNoGo > correctGo	MNI	go/no-go	Table 3
Lorenz et al., 2015	38	Corect Stop > Go	MNI	stop signal task	SM Table 1
Marco-Pallarés et al., 2008	10	correct Stop > correct Go	MNI	stop signal task	Table 1
Mazzola-Pomietto et al., 2009	16	correct No-Go > Correct Go	TAL	go/no-go	Table 3
Mohammadi et al., 2015	17	Correct Stop > Go	MNI	stop signal task	SM Table 2
Montojo et al., 2013	30	Correct Stop > Correct Go	MNI	stop signal task	Table 2
Nakata et al., 2008	15	Correct NoGo > Correct Go	TAL	go/no-go	Table 5
O'Connor et al., 2012	18	Correct NoGo > active baseline	MNI	go/no-go	Table 1
Rae et al., 2014	17	Stop specified correct > go specified correct	MNI	stop signal task	SM Table 2
		Stop select correct > go select correct		stop signal task	SM Table 2
Rodriguez-Pujadas et al., 2014	33	Correct Stop > Correct Go	MNI*	stop signal task	Table 2
Rothmayr et al., 2011	12	correct No-Go > Correct Go	MNI	go/no-go	Table 2
Rubia et al., 2006_1	21	successful NoGo > successful Go	TAL	go/no-go	Table 2

Rubia et al., 2006_2	25	successful no-go > successful go	TAL	go/no-go	Table 2
Schel et al., 2014	24	Correct Stop > Correct Go	MNI	stop signal task	Table 4
Sebastian et al., 2012	24	Correct NoGo > Correct Go	MNI	go/no-go	Table 5
		Correct Stop > Correct Go		stop signal task	Table 5
Sebastian et al., 2013a	48	Correct NoGo > active baseline including go	MNI	go/no-go	SM Table 1
		Correct Stop > Correct Go		stop signal task	SM Table 1
Sebastian et al., 2013b_1	24	Correct NoGo > active baseline including go	MNI	go/no-go	Table 3
		Correct Stop versus correct Go		stop signal task	Table 3
Sebastian et al., 2013b_2	21	correct NoGo > correct congruent go	MNI	go/no-go_hybrid response interference	Table 2
		Correct Stop versus correct congruent Go		stop signal task_hybrid response interference	Table 2
Sebastian et al., 2017	80	Correct Stop > Correct Go	MNI	stop signal task	from authors
Sharp et al., 2010	26	Correct Stop > Correct Go	MNI	stop signal task	SM Table 1
Steele et al. 2014	102	Correct NoGo > Correct Go	MNI	go/no-go	Table 2
Swann et al. 2012	16	Correct Stop > Correct Go	MNI	stop signal task	SM Table 2
Tabu et al., 2011	13	Correct Stop > Correct Go	MNI	stop signal task	text, page 280

Tabu et al., 2012	13	Correct Stop > Correct Go	MNI	stop signal task	SM Table 1 (hand task)
		Correct Stop > Correct Go		stop signal task	SM Table 1 (foot task)
Van der Meer et al., 2013	19	Correct Stop > Correct Go	MNI	stop signal task	SM Table 3A
Van Eijk et al., 2015_1	18	Correct NoGo > Correct Go	MNI	go/no-go	Table 4
		Correct Stop > Correct Go		stop signal task	Table 4
van Eijk et al., 2015_2	25	Correct NoGo > Correct congruent Go	MNI	go/no-go_hybrid response interference	Table 5
		Correct stop > correct congruent go		stop signal task_hybrid response interference	Table 5
Walther et al., 2010	17	correct No-Go > Correct Go	MNI	go/no-go	Table 1
Wilbertz et al., 2014	49	Correct Stop > Go	MNI	stop signal task	SM Table 2
Xu et al., 2015	18	Correct Stop > Go	MNI	stop signal task	Table 2
Xu et al., 2017	21	Correct Stop > Correct Go	MNI*	stop signal task	SM Table 1
Xue et al., 2008	15	Correct Stop > Correct Go	MNI	stop signal task	SM Table 1 (manual)
		Correct Stop > Correct Go		stop signal task	SM Table 1 (letter naming)

Zandbelt and Vink, 2010	24	Correct Stop > Go	MNI	stop signal task	SM Table 3B
Zandbelt et al. 2011	22	Correct Stop > Go	MNI	stop signal task	SM Table 11
Zheng et al., 2008	18	Correct Stop > Go	TAL	go/no-go	Table 1
Lock et al., 2011	13	correct No-Go > Correct Go	TAL	go/no-go	Table 2
Nosarti et al., 2006	14	(Correct no-go – go) – (correct 'odd' (aka go acting as a control for low freq of nogo) – go)	TAL	go/no-go	In text pg. 268
Durston et al., 2006	11	Successful No-go > Go	MNI	go/no-go	Table 2
He et al., 2014	30	Correct No-go > Go	MNI	go/no-go	Table 3
Schulz et al., 2004	9	Correct nogo > Correct Go	TAL	go/no-go	Table 2
Lee et al., 2018	34	Correct No-go > Go	MNI	go/no-go	Table 3
Heitzeg et al., 2010	20	Correct No-go > Go	MNI	go/no-go	Table 2
Mulder et al., 2008	12	Correct No-go > Go	MNI	go/no-go	Table 2
White et al., 2014	1133	stop success > baseline	MNI	stop signal task	SM Table 3
Lim et al., 2015	27	Stop Success > Go	MNI	stop signal task	SM Table
Bennett et al., 2009	11	Correct No-go – Correct go	TAL	go/no-go	Table 2
Cascio et al., 2015	37	Correct No-Go > Correct Go	MNI	go/no-go	Table 3
Rubia et al., 2013	66	Stop Success > Go	TAL	stop signal task	Table 2

Failure of Inhibition Database					
Author	N	contrast	space	paradigm	source of data
Boecker et al., 2011	15	failed stop - Go	MNI	stop signal task	SM Table 2
Boehler et al., 2010	15	unsuccessful stop > go	MNI	stop signal task	SM Table 4 and 3
Chen et al., 2015	25	failed nogo - go	MNI	go/no-go	Table 2
Chevrier et al., 2007	14	unsuccessful stop vs baseline	TAL	stop signal task	Table 1
Dambacher et al., 2015	15	False alarm > go	TAL	go/no-go	Table 1
Garavan et al., 2002	14	error vs. Baseline	TAL	go/no-go	Table 1
Garavan et al., 2003	16	error vs. Baseline	TAL	go/no-go	Table 1
Harle, 2016	34	stop error > go	TAL	stop signal task	SM Table 3
Hester et al., 2004	15	error vs. Baseline	TAL	go/no-go	Table 2
Hester et al., 2005	13	error vs correct go	TAL	go/no-go	Table 1
Hough et al., 2015	22	unsuccessful inhibition vs baseline	MNI	go/no-go	SM Table 5
Hsu et al., 2017	20	unsuccessful inhibition vs go	MNI	go/no-go	Table 3
Hughes et al., 2012	10	stop failure vs baseline	MNI	stop signal task	Table 4
Hughes et al., 2013	15	stop failure vs baseline	MNI	stop signal task	Table 3

Jahfari, 2011	20	failed stop > go	MNI	stop signal task	Table 4
Jahfari, 2012	16	failed stop > go	MNI	stop signal task	Table 5
Kiehl et al., 2000	14	error vs baseline (error of comission)	MNI	go/no-go	Table 1
Ko et al. 2014	23	unsuccessful inhibition - go	MNI	go/no-go	Table 3
Mohammadi et al., 2015	17	unsuccessful stop > go	MNI	stop signal task	SM Table 3
Rubia et al., 2003	20	unsuccessful stop vs go	TAL	stop signal task	Table 1
Sharp et al., 2010	26	incorrect stop vs correct go	MNI	stop signal task	SM Table 4
Steele et al., 2014	102	error vs correct go	MNI	go/no-go	Table 1 and 3
Xu et al., 2017	21	failed stop > go	TAL	stop signal task	SM Table 2
White et al., 2014	1133	stop failure > baseline	MNI	stop signal task	SM Table 3
Lim et al., 2015	27	stop failure > go	MNI	stop signal task	SM Table
Halari et al., 2009	21	stop failure – go	TAL	stop signal task	Table 3

References: Delay Consequence Sensitivity

Albrecht, K., Volz, K. G., Sutter, M., & Cramon, D. Y. von. (2013). What Do I Want and When Do I Want It: Brain Correlates of Decisions Made for Self and Other. PLOS ONE, 8(8), e73531. doi: 10.1371/journal.pone.0073531

Albrecht, K., Volz, K. G., Sutter, M., Laibson, D. I., & von Cramon, D. Y. (2011). What is for me is not for you: Brain correlates of intertemporal choice for self and other. Social Cognitive and Affective Neuroscience, 6(2), 218–225. doi: 10.1093/scan/nsq046

Banich, M. T., De La Vega, A., Andrews-Hanna, J. R., Mackiewicz Seghete, K., Du, Y., & Claus, E. D. (2013). Developmental trends and individual differences in brain systems involved in intertemporal choice during adolescence. Psychology of Addictive Behaviors, 27(2), 416–430. doi: 10.1037/a0031991

Bos, W. van den, Rodriguez, C. A., Schweitzer, J. B., & McClure, S. M. (2014). Connectivity Strength of Dissociable Striatal Tracts Predict Individual Differences in Temporal Discounting. Journal of Neuroscience, 34(31), 10298–10310. doi: 10.1523/JNEUROSCI.4105-13.2014

Castrellon, J. J., Young, J. S., Dang, L. C., Cowan, R. L., Zald, D. H., & Samanez-Larkin, G. R. (2019). Mesolimbic dopamine D2 receptors and neural representations of subjective value. Scientific Reports, 9(1), 20229. doi: 10.1038/s41598-019-56858-1

Christakou, A., Brammer, M., & Rubia, K. (2011). Maturation of limbic corticostriatal activation and connectivity associated with developmental changes in temporal discounting. NeuroImage, 54(2), 1344–1354. doi: 10.1016/j.neuroimage.2010.08.067

Cox, K. M., & Kable, J. W. (2014). BOLD Subjective Value Signals Exhibit Robust Range Adaptation. Journal of Neuroscience, 34(49), 16533–16543. doi: 10.1523/JNEUROSCI.3927-14.2014

de Water, E., Mies, G. W., Figner, B., Yoncheva, Y., van den Bos, W., Castellanos, F. X., … Scheres, A. (2017). Neural mechanisms of individual differences in temporal discounting of monetary and primary rewards in adolescents. NeuroImage, 153, 198–210. doi: 10.1016/j.neuroimage.2017.04.013

Deshpande, H. U., Mellis, A. M., Lisinski, J. M., Stein, J. S., Koffarnus, M. N., Paluch, R., … Bickel, W. K. (2019). Reinforcer pathology: Common neural substrates for delay discounting and snack purchasing in prediabetics. Brain and Cognition, 132, 80–88. doi: 10.1016/j.bandc.2019.03.003

Elton, A., Smith, C. T., Parrish, M. H., & Boettiger, C. A. (2017). Neural Systems Underlying Individual Differences in Intertemporal Decision-making. Journal of Cognitive Neuroscience, 29(3), 467–479. doi: 10.1162/jocn_a_01069

Eppinger, B., Heekeren, H. R., & Li, S.-C. (2018). Age differences in the neural mechanisms of intertemporal choice under subjective decision conflict. Cerebral Cortex, 28(11), 3764–3774. doi: 10.1093/cercor/bhx239

Eppinger, B., Nystrom, L. E., & Cohen, J. D. (2012). Reduced Sensitivity to Immediate Reward during Decision-Making in Older than Younger Adults. PLOS ONE, 7(5), e36953. doi: 10.1371/journal.pone.0036953

Faralla, V., Benuzzi, F., Lui, F., Baraldi, P., Dimitri, N., & Nichelli, P. (2015). Neural correlates in intertemporal choice of gains and losses. Journal of Neuroscience, Psychology, and Economics, 8(1), 27–47. doi: 10.1037/npe0000032

Hamilton, K. R., Smith, J. F., Gonçalves, S. F., Nketia, J. A., Tasheuras, O. N., Yoon, M., … Shackman, A. J. (2020). Striatal bases of temporal discounting in early adolescents. Neuropsychologia, 144, 107492. doi: 10.1016/j.neuropsychologia.2020.107492

Hare, T., Hakimi, S., & Rangel, A. (2014). Activity in dlPFC and its effective connectivity to vmPFC are associated with temporal discounting. Frontiers in Neuroscience, 8. Retrieved from https://www.frontiersin.org/articles/10.3389/fnins.2014.00050

Hill, P. F., Yi, R., Spreng, R. N., & Diana, R. A. (2017). Neural congruence between intertemporal and interpersonal self-control: Evidence from delay and social discounting. NeuroImage, 162, 186–198. doi: 10.1016/j.neuroimage.2017.08.071

Jimura, K., Chushak, M. S., & Braver, T. S. (2013). Impulsivity and Self-Control during Intertemporal Decision Making Linked to the Neural Dynamics of Reward Value Representation. Journal of Neuroscience, 33(1), 344–357. doi: 10.1523/JNEUROSCI.0919-12.2013

Kable, J. W., & Glimcher, P. W. (2007). The neural correlates of subjective value during intertemporal choice. Nature Neuroscience, 10(12), 1625–1633. doi: 10.1038/nn2007

Kable, J. W., & Glimcher, P. W. (2010). An “As Soon As Possible” Effect in Human Intertemporal Decision Making: Behavioral Evidence and Neural Mechanisms. Journal of Neurophysiology, 103(5), 2513–2531. doi: 10.1152/jn.00177.2009

King, J. A., Geisler, D., Bernardoni, F., Ritschel, F., Böhm, I., Seidel, M., … Ehrlich, S. (2016). Altered Neural Efficiency of Decision Making During Temporal Reward Discounting in Anorexia Nervosa. Journal of the American Academy of Child & Adolescent Psychiatry, 55(11), 972–979. doi: 10.1016/j.jaac.2016.08.005

Laube, C., Lorenz, R., & van den Bos, W. (2020). Pubertal testosterone correlates with adolescent impatience and dorsal striatal activity. Developmental Cognitive Neuroscience, 42, 100749. doi: 10.1016/j.dcn.2019.100749

Lempert, K. M., Speer, M. E., Delgado, M. R., & Phelps, E. A. (2017). Positive autobiographical memory retrieval reduces temporal discounting. Social Cognitive and Affective Neuroscience, 12(10), 1584–1593. doi: 10.1093/scan/nsx086

Liu, L., Feng, T., Wang, J., & Li, H. (2012). The neural dissociation of subjective valuation from choice processes in intertemporal choice. Behavioural Brain Research, 231(1), 40–47. doi: 10.1016/j.bbr.2012.02.045

Luo, S., Ainslie, G., Giragosian, L., & Monterosso, J. R. (2009). Behavioral and Neural Evidence of Incentive Bias for Immediate Rewards Relative to Preference-Matched Delayed

Rewards. Journal of Neuroscience, 29(47), 14820–14827. doi: 10.1523/JNEUROSCI.4261-09.2009

Luo, S., Ainslie, G., Pollini, D., Giragosian, L., & Monterosso, J. R. (2012). Moderators of the association between brain activation and farsighted choice. NeuroImage, 59(2), 1469–1477. doi: 10.1016/j.neuroimage.2011.08.004

Massar, S. A. A., Libedinsky, C., Weiyan, C., Huettel, S. A., & Chee, M. W. L. (2015). Separate and overlapping brain areas encode subjective value during delay and effort discounting. NeuroImage, 120, 104–113. doi: 10.1016/j.neuroimage.2015.06.080

Mavrogiorgou, P., Enzi, B., Klimm, A.-K., Köhler, E., Roser, P., Norra, C., & Juckel, G. (2017). Serotonergic modulation of orbitofrontal activity and its relevance for decision making and impulsivity. Human Brain Mapping, 38(3), 1507–1517. doi: 10.1002/hbm.23468

McClure, S. M., Laibson, D. I., Loewenstein, G., & Cohen, J. D. (2004). Separate Neural Systems Value Immediate and Delayed Monetary Rewards. Science, 306(5695), 503–507. doi: 10.1126/science.1100907

Miedl, S. F., Wiswede, D., Marco-Pallarés, J., Ye, Z., Fehr, T., Herrmann, M., & Münte, T. F. (2015). The neural basis of impulsive discounting in pathological gamblers. Brain Imaging and Behavior, 9(4), 887–898. doi: 10.1007/s11682-015-9352-1

Murawski, C., Harris, P. G., Bode, S., D, J. F. D., & Egan, G. F. (2012). Led into Temptation? Rewarding Brand Logos Bias the Neural Encoding of Incidental Economic Decisions. PLOS ONE, 7(3), e34155. doi: 10.1371/journal.pone.0034155

Norman, L. J., Carlisi, C. O., Christakou, A., Chantiluke, K., Murphy, C., Simmons, A., … Rubia, K. (2017). Neural dysfunction during temporal discounting in paediatric Attention-Deficit/Hyperactivity Disorder and Obsessive-Compulsive Disorder. Psychiatry Research: Neuroimaging, 269, 97–105. doi: 10.1016/j.pscychresns.2017.09.008

O'Connell, G., Hsu, C.-T., Christakou, A., & Chakrabarti, B. (2018). Thinking about others and the future: Neural correlates of perspective taking relate to preferences for delayed rewards. Cognitive, Affective, & Behavioral Neuroscience, 18(1), 35–42. doi: 10.3758/s13415-017-0550-8

Peters, J., & Büchel, C. (2010). Episodic Future Thinking Reduces Reward Delay Discounting through an Enhancement of Prefrontal-Mediotemporal Interactions. Neuron, 66(1), 138–148. doi: 10.1016/j.neuron.2010.03.026

Pine, A., Seymour, B., Roiser, J. P., Bossaerts, P., Friston, K. J., Curran, H. V., & Dolan, R. J. (2009). Encoding of Marginal Utility across Time in the Human Brain. Journal of Neuroscience, 29(30), 9575–9581. doi: 10.1523/JNEUROSCI.1126-09.2009

Prévost, C., Pessiglione, M., Météreau, E., Cléry-Melin, M.-L., & Dreher, J.-C. (2010). Separate Valuation Subsystems for Delay and Effort Decision Costs. Journal of Neuroscience, 30(42), 14080–14090. doi: 10.1523/JNEUROSCI.2752-10.2010

Ripke, S., Hübner, T., Mennigen, E., Müller, K. U., Rodehacke, S., Schmidt, D., … Smolka, M. N. (2012). Reward processing and intertemporal decision making in adults and

adolescents: The role of impulsivity and decision consistency. Brain Research, 1478, 36–47. doi: 10.1016/j.brainres.2012.08.034

Samanez-Larkin, G., Mata, R., Radu, P., Ballard, I., Carstensen, L., & McClure, S. (2011). Age Differences in Striatal Delay Sensitivity during Intertemporal Choice in Healthy Adults. Frontiers in Neuroscience, 5. Retrieved from https://www.frontiersin.org/articles/10.3389/fnins.2011.00126

Sasse, L. K., Peters, J., & Brassen, S. (2017). Cognitive Control Modulates Effects of Episodic Simulation on Delay Discounting in Aging. Frontiers in Aging Neuroscience, 9. Retrieved from https://www.frontiersin.org/articles/10.3389/fnagi.2017.00058

Seaman, K. L., Brooks, N., Karrer, T. M., Castrellon, J. J., Perkins, S. F., Dang, L. C., … Samanez-Larkin, G. R. (2018). Subjective value representations during effort, probability and time discounting across adulthood. Social Cognitive and Affective Neuroscience, 13(5), 449–459. doi: 10.1093/scan/nsy021

Sripada, C. S., Gonzalez, R., Phan, K. L., & Liberzon, I. (2011). The neural correlates of intertemporal decision-making: Contributions of subjective value, stimulus type, and trait impulsivity. Human Brain Mapping, 32(10), 1637–1648. doi: https://doi.org/10.1002/hbm.21136

Waegeman, A., Declerck, C. H., Boone, C., Van Hecke, W., & Parizel, P. M. (2014). Individual differences in self-control in a time discounting task: An fMRI study. Journal of Neuroscience, Psychology, and Economics, 7(2), 65–79. doi: 10.1037/npe0000018

Wang, Y., Hu, Y., Xu, J., Zhou, H., Lin, X., Du, X., & Dong, G. (2017). Dysfunctional Prefrontal Function Is Associated with Impulsivity in People with Internet Gaming Disorder during a Delay Discounting Task. Frontiers in Psychiatry, 8. Retrieved from https://www.frontiersin.org/articles/10.3389/fpsyt.2017.00287

Wiehler, A., Petzschner, F. H., Stephan, K. E., & Peters, J. (2017). Episodic Tags Enhance Striatal Valuation Signals during Temporal Discounting in pathological Gamblers. ENeuro, 4(3). doi: 10.1523/ENEURO.0159-17.2017

Wittmann, M., Leland, D. S., & Paulus, M. P. (2007). Time and decision making: Differential contribution of the posterior insular cortex and the striatum during a delay discounting task. Experimental Brain Research, 179(4), 643–653. doi: 10.1007/s00221-006-0822-y

Wittmann, M., Lovero, K. L., Lane, S. D., & Paulus, M. P. (2010). Now or later? Striatum and insula activation to immediate versus delayed rewards. Journal of Neuroscience, Psychology, and Economics, 3(1), 15–26. doi: 10.1037/a0017252

Xu, L., Liang, Z.-Y., Wang, K., Li, S., & Jiang, T. (2009). Neural mechanism of intertemporal choice: From discounting future gains to future losses. Brain Research, 1261, 65–74. doi: 10.1016/j.brainres.2008.12.061

Zhuang, J.-Y., Wang, J.-X., Lei, Q., Zhang, W., & Fan, M. (2020). Neural Basis of Increased Cognitive Control of Impulsivity During the Mid-Luteal Phase Relative to the Late Follicular Phase of the Menstrual Cycle. Frontiers in Human Neuroscience, 14. Retrieved from https://www.frontiersin.org/articles/10.3389/fnhum.2020.568399

References: Response Inhibition

Aron, A. R., & Poldrack, R. A. (2006). Cortical and subcortical contributions to Stop signal response inhibition: Role of the subthalamic nucleus. J Neurosci, 26(9), 2424–2433. doi: 10.1523/JNEUROSCI.4682-05.2006

Bennett, D. S., Mohamed, F. B., Carmody, D. P., Bendersky, M., Patel, S., Khorrami, M., … Lewis, M. (2009). Response inhibition among early adolescents prenatally exposed to tobacco: An fMRI study. Neurotoxicology and Teratology, 31(5), 283–290. doi: 10.1016/j.ntt.2009.03.003

Berkman, E. T., Kahn, L. E., & Merchant, J. S. (2014). Training-induced changes in inhibitory control network activity. J Neurosci, 34(1), 149–157. doi: 10.1523/JNEUROSCI.3564-13.2014

Bobb, D. S., Adinoff, B., Laken, S. J., McClintock, S. M., Rubia, K., Huang, H. W., … Kozel, F. A. (2012). Neural correlates of successful response inhibition in unmedicated patients with late-life depression. Am J Geriatr Psychiatry, 20(12), 1057–1069. doi: 10.1097/JGP.0b013e318235b728

Boecker, M., Drueke, B., Vorhold, V., Knops, A., Philippen, B., & Gauggel, S. (2011). When Response Inhibition is Followed by Response Reengagement: An Event-Related fMRI Study. Human Brain Mapping, 32(1), 94–106. doi: 10.1002/hbm.21001

Boehler, C. N., Appelbaum, L. G., Krebs, R. M., Hopf, J. M., & Woldorff, M. G. (2010). Pinning down response inhibition in the brain—Conjunction analyses of the Stop-signal task. Neuroimage, 52(4), 1621–1632. doi: 10.1016/j.neuroimage.2010.04.276

Cai, W., Cannistraci, C. J., Gore, J. C., & Leung, H. C. (2014). Sensorimotor-independent prefrontal activity during response inhibition. Hum Brain Mapp, 35(5), 2119–2136. doi: 10.1002/hbm.22315

Cai, W., & Leung, H. C. (2009). Cortical activity during manual response inhibition guided by color and orientation cues. Brain Res, 1261, 20–28. doi: 10.1016/j.brainres.2008.12.073

Cai, W., & Leung, H. C. (2011). Rule-guided executive control of response inhibition: Functional topography of the inferior frontal cortex. PLoS One, 6(6), e20840. doi: 10.1371/journal.pone.0020840

Cascio, C. N., Carp, J., O'Donnell, M. B., Tinney, F. J., Jr., Bingham, C. R., Shope, J. T., … Falk, E. B. (2015). Buffering Social Influence: Neural Correlates of Response Inhibition Predict Driving Safety in the Presence of a Peer. Journal of Cognitive Neuroscience, 27(1), 83–95. doi: 10.1162/jocn_a_00693

Chen, C.-Y., Yen, J.-Y., Yen, C.-F., Chen, C.-S., Liu, G.-C., Liang, C.-Y., & Ko, C.-H. (2015). Aberrant brain activation of error processing among adults with attention deficit and hyperactivity disorder. The Kaohsiung Journal of Medical Sciences, 31(4), 179–187. doi: 10.1016/j.kjms.2015.01.001

Chevrier, A. D., Noseworthy, M. D., & Schachar, R. (2007). Dissociation of response inhibition and performance monitoring in the stop signal task using event-related fMRI. Hum Brain Mapp, 28(12), 1347–1358. doi: 10.1002/hbm.20355

Chikara, R. K., Chang, E. C., Lu, Y. C., Lin, D. S., Lin, C. T., & Ko, L. W. (2018). Monetary Reward and Punishment to Response Inhibition Modulate Activation and Synchronization Within the Inhibitory Brain Network. Front Hum Neurosci, 12, 27. doi: 10.3389/fnhum.2018.00027

Chikazoe, J., Jimura, K., Asari, T., Yamashita, K., Morimoto, H., Hirose, S., … Konishi, S. (2009). Functional dissociation in right inferior frontal cortex during performance of go/no-go task. Cereb Cortex, 19(1), 146–152. doi: 10.1093/cercor/bhn065

Chikazoe, J., Jimura, K., Hirose, S., Yamashita, K., Miyashita, Y., & Konishi, S. (2009). Preparation to inhibit a response complements response inhibition during performance of a stop-signal task. J Neurosci, 29(50), 15870–15877. doi: 10.1523/JNEUROSCI.3645-09.2009

Congdon, E., Altshuler, L. L., Mumford, J. A., Karlsgodt, K. H., Sabb, F. W., Ventura, J., … Poldrack, R. A. (2014). Neural activation during response inhibition in adult attention-deficit/hyperactivity disorder: Preliminary findings on the effects of medication and symptom severity. Psychiatry Res, 222(1–2), 17–28. doi: 10.1016/j.pscychresns.2014.02.002

Coxon, J. P., Goble, D. J., Leunissen, I., Van Impe, A., Wenderoth, N., & Swinnen, S. P. (2016). Functional Brain Activation Associated with Inhibitory Control Deficits in Older Adults. Cereb Cortex, 26(1), 12–22. doi: 10.1093/cercor/bhu165

Czapla, M., Baeuchl, C., Simon, J. J., Richter, B., Kluge, M., Friederich, H. C., … Loeber, S. (2017). Do alcohol-dependent patients show different neural activation during response inhibition than healthy controls in an alcohol-related fMRI go/no-go-task? Psychopharmacology (Berl), 234(6), 1001–1015. doi: 10.1007/s00213-017-4541-9

Dambacher, F., Sack, A. T., Lobbestael, J., Arntz, A., Brugman, S., & Schuhmann, T. (2015). Out of control: Evidence for anterior insula involvement in motor impulsivity and reactive aggression. Social Cognitive and Affective Neuroscience, 10(4), 508–516. doi: 10.1093/scan/nsu077

Durston, S., Mulder, M., Casey, B. J., Ziermans, T., & van Engeland, H. (2006). Activation in ventral prefrontal cortex is sensitive to genetic vulnerability for attention-deficit hyperactivity disorder. Biological Psychiatry, 60(10), 1062–1070. doi: 10.1016/j.biopsych.2005.12.020

Fassbender, C., Murphy, K., Foxe, J. J., Wylie, G. R., Javitt, D. C., Robertson, I. H., & Garavan, H. (2004). A topography of executive functions and their interactions revealed by functional magnetic resonance imaging. Cognitive Brain Research, 20(2), 132–143. doi: 10.1016/j.cogbrainres.2004.02.007

Fauth-Buhler, M., de Rover, M., Rubia, K., Garavan, H., Abbott, S., Clark, L., … Robbins, T. W. (2012). Brain networks subserving fixed versus performance-adjusted delay stop trials in a stop signal task. Behav Brain Res, 235(1), 89–97. doi: 10.1016/j.bbr.2012.07.023

Fedota, J. R., Hardee, J. E., Perez-Edgar, K., & Thompson, J. C. (2014). Representation of response alternatives in human presupplementary motor area: Multi-voxel pattern analysis in a go/no-go task. Neuropsychologia, 56, 110–118. doi: 10.1016/j.neuropsychologia.2013.12.022

Fuentes-Claramonte, P., Avila, C., Rodriguez-Pujadas, A., Costumero, V., Ventura-Campos, N., Bustamante, J. C., … Barros-Loscertales, A. (2016). Inferior frontal cortex activity is modulated by reward sensitivity and performance variability. Biol Psychol, 114, 127–137. doi: 10.1016/j.biopsycho.2016.01.001

Ganos, C., Kuhn, S., Kahl, U., Schunke, O., Feldheim, J., Gerloff, C., … Munchau, A. (2014). Action inhibition in Tourette syndrome. Mov Disord, 29(12), 1532–1538. doi: 10.1002/mds.25944

Garavan, H., Ross, T. J., Kaufman, J., & Stein, E. A. (2003). A midline dissociation between error-processing and response-conflict monitoring. Neuroimage, 20(2), 1132–1139. doi: 10.1016/S1053-8119(03)00334-3

Garavan, H., Ross, T. J., Murphy, K., Roche, R. A., & Stein, E. A. (2002). Dissociable executive functions in the dynamic control of behavior: Inhibition, error detection, and correction. Neuroimage, 17(4), 1820–1829. doi: 10.1006/nimg.2002.1326

Garavan, H., Ross, T. J., & Stein, E. A. (1999). Right hemispheric dominance of inhibitory control: An event-related functional MRI study. Proc Natl Acad Sci U S A, 96(14), 8301–8306. doi: 10.1073/pnas.96.14.8301

Geng, J. J., Ruff, C. C., & Driver, J. (2009). Saccades to a remembered location elicit spatially specific activation in human retinotopic visual cortex. J Cogn Neurosci, 21(2), 230–245. doi: 10.1162/jocn.2008.21025

Ghahremani, D. G., Lee, B., Robertson, C. L., Tabibnia, G., Morgan, A. T., De Shetler, N., … London, E. D. (2012). Striatal dopamine D(2)/D(3) receptors mediate response inhibition and related activity in frontostriatal neural circuitry in humans. J Neurosci, 32(21), 7316–7324. doi: 10.1523/JNEUROSCI.4284-11.2012

Halari, R., Simic, M., Pariante, C. M., Papadopoulos, A., Cleare, A., Brammer, M., … Rubia, K. (2009). Reduced activation in lateral prefrontal cortex and anterior cingulate during attention and cognitive control functions in medication-naïve adolescents with depression compared to controls. Journal of Child Psychology and Psychiatry, 50(3), 307–316. doi: 10.1111/j.1469-7610.2008.01972.x

Harle, K. M., Zhang, S., Ma, N., Yu, A. J., & Paulus, M. P. (2016). Reduced Neural Recruitment for Bayesian Adjustment of Inhibitory Control in Methamphetamine Dependence. Biol Psychiatry Cogn Neurosci Neuroimaging, 1(5), 448–459. doi: 10.1016/j.bpsc.2016.06.008

He, Q., Xiao, L., Xue, G., Wong, S., Ames, S. L., Schembre, S. M., & Bechara, A. (2014). Poor ability to resist tempting calorie rich food is linked to altered balance between neural systems involved in urge and self-control. Nutrition Journal, 13(1), 92. doi: 10.1186/1475-2891-13-92

Heitzeg, M. M., Nigg, J. T., Yau, W.-Y. W., Zucker, R. A., & Zubieta, J.-K. (2010). Striatal dysfunction marks preexisting risk and medial prefrontal dysfunction is related to problem drinking in children of alcoholics. Biological Psychiatry, 68(3), 287–295. doi: 10.1016/j.biopsych.2010.02.020

Hester, R., Foxe, J. J., Molholm, S., Shpaner, M., & Garavan, H. (2005). Neural mechanisms involved in error processing: A comparison of errors made with and without awareness. Neuroimage, 27(3), 602–608. doi: 10.1016/j.neuroimage.2005.04.035

Hester, R. L., Murphy, K., Foxe, J. J., Foxe, D. M., Javitt, D. C., & Garavan, H. (2004). Predicting success: Patterns of cortical activation and deactivation prior to response inhibition. J Cogn Neurosci, 16(5), 776–785. doi: 10.1162/089892904970726

Hester, R., Murphy, K., & Garavan, H. (2004). Beyond common resources: The cortical basis for resolving task interference. Neuroimage, 23(1), 202–212. doi: 10.1016/j.neuroimage.2004.05.024

Hough, C. M., Luks, T. L., Lai, K., Vigil, O., Guillory, S., Nongpiur, A., … Mathews, C. A. (2016). Comparison of brain activation patterns during executive function tasks in hoarding disorder and non-hoarding OCD. Psychiatry Res Neuroimaging, 255, 50–59. doi: 10.1016/j.pscychresns.2016.07.007

Hsu, J. S., Wang, P. W., Ko, C. H., Hsieh, T. J., Chen, C. Y., & Yen, J. Y. (2017). Altered brain correlates of response inhibition and error processing in females with obesity and sweet food addiction: A functional magnetic imaging study. Obes Res Clin Pract, 11(6), 677–686. doi: 10.1016/j.orcp.2017.04.011

Hughes, M. E., Fulham, W. R., Johnston, P. J., & Michie, P. T. (2012). Stop-signal response inhibition in schizophrenia: Behavioural, event-related potential and functional neuroimaging data. Biol Psychol, 89(1), 220–231. doi: 10.1016/j.biopsycho.2011.10.013

Hughes, M. E., Johnston, P. J., Fulham, W. R., Budd, T. W., & Michie, P. T. (2013). Stop-signal task difficulty and the right inferior frontal gyrus. Behav Brain Res, 256, 205–213. doi: 10.1016/j.bbr.2013.08.026

Jahfari, S., Verbruggen, F., Frank, M. J., Waldorp, L. J., Colzato, L., Ridderinkhof, K. R., & Forstmann, B. U. (2012). How preparation changes the need for top-down control of the basal ganglia when inhibiting premature actions. J Neurosci, 32(32), 10870–10878. doi: 10.1523/JNEUROSCI.0902-12.2012

Jahfari, S., Waldorp, L., Ridderinkhof, K. R., & Scholte, H. S. (2015). Visual information shapes the dynamics of corticobasal ganglia pathways during response selection and inhibition. J Cogn Neurosci, 27(7), 1344–1359. doi: 10.1162/jocn_a_00792

Jahfari, S., Waldorp, L., van den Wildenberg, W. P., Scholte, H. S., Ridderinkhof, K. R., & Forstmann, B. U. (2011). Effective connectivity reveals important roles for both the hyperdirect (fronto-subthalamic) and the indirect (fronto-striatal-pallidal) fronto-basal ganglia pathways during response inhibition. J Neurosci, 31(18), 6891–6899. doi: 10.1523/JNEUROSCI.5253-10.2011

Kaladjian, A., Jeanningros, R., Azorin, J. M., Grimault, S., Anton, J. L., & Mazzola-Pomietto, P. (2007). Blunted activation in right ventrolateral prefrontal cortex during motor response inhibition in schizophrenia. Schizophrenia Research, 97(1–3), 184–193. doi: 10.1016/j.schres.2007.07.033

Kaladjian, A., Jeanningros, R., Azorin, J. M., Nazarian, B., Roth, M., Anton, J. L., & Mazzola-Pomietto, P. (2009). Remission from mania is associated with a decrease in amygdala activation during motor response inhibition. Bipolar Disord, 11(5), 530–538. doi: 10.1111/j.1399-5618.2009.00722.x

Kaladjian, A., Jeanningros, R., Azorin, J. M., Nazarian, B., Roth, M., & Mazzola-Pomietto, P. (2009). Reduced brain activation in euthymic bipolar patients during response inhibition: An event-related fMRI study. Psychiatry Res, 173(1), 45–51. doi: 10.1016/j.pscychresns.2008.08.003

Kelly, A. M., Hester, R., Murphy, K., Javitt, D. C., Foxe, J. J., & Garavan, H. (2004). Prefrontal-subcortical dissociations underlying inhibitory control revealed by event-related fMRI. Eur J Neurosci, 19(11), 3105–3112. doi: 10.1111/j.0953-816X.2004.03429.x

Kenner, N. M., Mumford, J. A., Hommer, R. E., Skup, M., Leibenluft, E., & Poldrack, R. A. (2010). Inhibitory motor control in response stopping and response switching. J Neurosci, 30(25), 8512–8518. doi: 10.1523/JNEUROSCI.1096-10.2010

Kiehl, K. A., Liddle, P. F., & Hopfinger, J. B. (2000). Error processing and the rostral anterior cingulate: An event-related fMRI study. Psychophysiology, 37(2), 216–223.

Ko, C. H., Hsieh, T. J., Chen, C. Y., Yen, C. F., Chen, C. S., Yen, J. Y., … Liu, G. C. (2014). Altered brain activation during response inhibition and error processing in subjects with Internet gaming disorder: A functional magnetic imaging study. Eur Arch Psychiatry Clin Neurosci, 264(8), 661–672. doi: 10.1007/s00406-013-0483-3

Ko, L.-W. ; S. (2016). Neural mechanisms of inhibitory response in a battlefield scenario: A silmutaneous fMRI-EEG study. Frontiers in Human Neuroscience, 10, 1–15.

Kohler, S., Schumann, A., de la Cruz, F., Wagner, G., & Bar, K. J. (2018). Towards response success prediction: An integrative approach using high-resolution fMRI and autonomic indices. Neuropsychologia, 119, 182–190. doi: 10.1016/j.neuropsychologia.2018.08.003

Lavallee, C. F., Herrmann, C. S., Weerda, R., & Huster, R. J. (2014). Stimulus-response mappings shape inhibition processes: A combined EEG-fMRI study of contextual stopping. PLoS One, 9(4), e96159. doi: 10.1371/journal.pone.0096159

Lee, N. C., Weeda, W. D., Insel, C., Somerville, L. H., Krabbendam, L., & Huizinga, M. (2018). Neural substrates of the influence of emotional cues on cognitive control in risk-taking adolescents. Developmental Cognitive Neuroscience, 31, 20–34. doi: 10.1016/j.dcn.2018.04.007

Liddle, P. F., Kiehl, K. A., & Smith, A. M. (2001). Event-related fMRI study of response inhibition. Hum Brain Mapp, 12(2), 100–109.

Lim, L., Hart, H., Mehta, M. A., Simmons, A., Mirza, K., & Rubia, K. (2015). Neural Correlates of Error Processing in Young People With a History of Severe Childhood Abuse: An fMRI Study. American Journal of Psychiatry, 172(9), 892–900. doi: 10.1176/appi.ajp.2015.14081042

Lock, J., Garrett, A., Beenhakker, J., & Reiss, A. L. (2011). Aberrant brain activation during a response inhibition task in adolescent eating disorder subtypes. The American Journal of Psychiatry, 168(1), 55–64. doi: 10.1176/appi.ajp.2010.10010056

Lorenz, R. C., Gleich, T., Buchert, R., Schlagenhauf, F., Kuhn, S., & Gallinat, J. (2015). Interactions between glutamate, dopamine, and the neuronal signature of response inhibition in the human striatum. Hum Brain Mapp, 36(10), 4031–4040. doi: 10.1002/hbm.22895

Marco-Pallares, J., Camara, E., Munte, T. F., & Rodriguez-Fornells, A. (2008). Neural mechanisms underlying adaptive actions after slips. J Cogn Neurosci, 20(9), 1595–1610. doi: 10.1162/jocn.2008.20117

Mazzola-Pomietto, P., Kaladjian, A., Azorin, J. M., Anton, J. L., & Jeanningros, R. (2009). Bilateral decrease in ventrolateral prefrontal cortex activation during motor response inhibition in mania. J Psychiatr Res, 43(4), 432–441. doi: 10.1016/j.jpsychires.2008.05.004

Mohammadi, B., Kollewe, K., Cole, D. M., Fellbrich, A., Heldmann, M., Samii, A., … Kramer, U. M. (2015). Amyotrophic lateral sclerosis affects cortical and subcortical activity underlying motor inhibition and action monitoring. Hum Brain Mapp, 36(8), 2878–2889. doi: 10.1002/hbm.22814

Montojo, C. A., Jalbrzikowski, M., Congdon, E., Domicoli, S., Chow, C., Dawson, C., … Bearden, C. E. (2015). Neural substrates of inhibitory control deficits in 22q11.2 deletion syndrome. Cereb Cortex, 25(4), 1069–1079. doi: 10.1093/cercor/bht304

Mulder, M. J., Baeyens, D., Davidson, M. C., Casey, B. J., DEN Ban, E. V., VAN Engeland, H., & Durston, S. (2008). Familial vulnerability to ADHD affects activity in the cerebellum in addition to the prefrontal systems. Journal of the American Academy of Child and Adolescent Psychiatry, 47(1), 68–75. doi: 10.1097/chi.0b013e31815a56dc

Nakata, H., Sakamoto, K., Ferretti, A., Gianni Perrucci, M., Del Gratta, C., Kakigi, R., & Romani, G. L. (2008). Executive functions with different motor outputs in somatosensory Go/Nogo tasks: An event-related functional MRI study. Brain Res Bull, 77(4), 197–205. doi: 10.1016/j.brainresbull.2008.07.008

Nosarti, C., Rubia, K., Smith, A. B., Frearson, S., Williams, S. C., Rifkin, L., & Murray, R. M. (2006). Altered functional neuroanatomy of response inhibition in adolescent males who were born very preterm. Developmental Medicine and Child Neurology, 48(4), 265–271. doi: 10.1017/S0012162206000582

O'Connor, D. A., Rossiter, S., Yucel, M., Lubman, D. I., & Hester, R. (2012). Successful inhibitory control over an immediate reward is associated with attentional disengagement in visual processing areas. Neuroimage, 62(3), 1841–1847. doi: 10.1016/j.neuroimage.2012.05.040

Rae, C. L., Hughes, L. E., Weaver, C., Anderson, M. C., & Rowe, J. B. (2014). Selection and stopping in voluntary action: A meta-analysis and combined fMRI study. Neuroimage, 86, 381–391. doi: 10.1016/j.neuroimage.2013.10.012

Rodriguez-Pujadas, A., Sanjuan, A., Fuentes, P., Ventura-Campos, N., Barros-Loscertales, A., & Avila, C. (2014). Differential neural control in early bilinguals and monolinguals during response inhibition. Brain Lang, 132, 43–51. doi: 10.1016/j.bandl.2014.03.003

Rothmayr, C., Sodian, B., Hajak, G., Dohnel, K., Meinhardt, J., & Sommer, M. (2011). Common and distinct neural networks for false-belief reasoning and inhibitory control. Neuroimage, 56(3), 1705–1713. doi: 10.1016/j.neuroimage.2010.12.052

Rubia, K., Smith, A. B., Brammer, M. J., & Taylor, E. (2003). Right inferior prefrontal cortex mediates response inhibition while mesial prefrontal cortex is responsible for error detection. Neuroimage, 20(1), 351–358. doi: 10.1016/s1053-8119(03)00275-1

Rubia, Katya, Lim, L., Ecker, C., Halari, R., Giampietro, V., Simmons, A., … Smith, A. (2013). Effects of age and gender on neural networks of motor response inhibition: From adolescence to mid-adulthood. NeuroImage, 83, 690–703. doi: 10.1016/j.neuroimage.2013.06.078

Rubia, Katya, Smith, A. B., Woolley, J., Nosarti, C., Heyman, I., Taylor, E., & Brammer, M. (2006). Progressive increase of frontostriatal brain activation from childhood to adulthood during event-related tasks of cognitive control. Human Brain Mapping, 27(12), 973–993. doi: 10.1002/hbm.20237

Schel, M. A., Kuhn, S., Brass, M., Haggard, P., Ridderinkhof, K. R., & Crone, E. A. (2014). Neural correlates of intentional and stimulus-driven inhibition: A comparison. Front Hum Neurosci, 8, 27. doi: 10.3389/fnhum.2014.00027

Schulz, K. P., Fan, J., Tang, C. Y., Newcorn, J. H., Buchsbaum, M. S., Cheung, A. M., & Halperin, J. M. (2004). Response Inhibition in Adolescents Diagnosed With Attention Deficit Hyperactivity Disorder During Childhood: An Event-Related fMRI Study. American Journal of Psychiatry, 161(9), 1650–1657. doi: 10.1176/appi.ajp.161.9.1650

Sebastian, A., Baldermann, C., Feige, B., Katzev, M., Scheller, E., Hellwig, B., … Kloppel, S. (2013). Differential effects of age on subcomponents of response inhibition. Neurobiol Aging, 34(9), 2183–2193. doi: 10.1016/j.neurobiolaging.2013.03.013

Sebastian, A., Gerdes, B., Feige, B., Kloppel, S., Lange, T., Philipsen, A., … Tuscher, O. (2012). Neural correlates of interference inhibition, action withholding and action cancelation in adult ADHD. Psychiatry Res, 202(2), 132–141. doi: 10.1016/j.pscychresns.2012.02.010

Sebastian, A., Pohl, M. F., Kloppel, S., Feige, B., Lange, T., Stahl, C., … Tuscher, O. (2013). Disentangling common and specific neural subprocesses of response inhibition. Neuroimage, 64, 601–615. doi: 10.1016/j.neuroimage.2012.09.020

Sebastian, A., Rossler, K., Wibral, M., Mobascher, A., Lieb, K., Jung, P., & Tuscher, O. (2017). Neural Architecture of Selective Stopping Strategies: Distinct Brain Activity Patterns Are Associated with Attentional Capture But Not with Outright Stopping. J Neurosci, 37(40), 9785–9794. doi: 10.1523/JNEUROSCI.1476-17.2017

Sharp, D. J., Bonnelle, V., De Boissezon, X., Beckmann, C. F., James, S. G., Patel, M. C., & Mehta, M. A. (2010). Distinct frontal systems for response inhibition, attentional capture, and error processing. Proc Natl Acad Sci U S A, 107(13), 6106–6111. doi: 10.1073/pnas.1000175107

Steele, V. R., Claus, E. D., Aharoni, E., Harenski, C., Calhoun, V. D., Pearlson, G., & Kiehl, K. A. (2014). A large scale (N=102) functional neuroimaging study of error processing in a Go/NoGo task. Behav Brain Res, 268, 127–138. doi: 10.1016/j.bbr.2014.04.001

Swann, N. C., Cai, W., Conner, C. R., Pieters, T. A., Claffey, M. P., George, J. S., … Tandon, N. (2012). Roles for the pre-supplementary motor area and the right inferior frontal gyrus in stopping action: Electrophysiological responses and functional and structural connectivity. Neuroimage, 59(3), 2860–2870. doi: 10.1016/j.neuroimage.2011.09.049

Tabu, H., Mima, T., Aso, T., Takahashi, R., & Fukuyama, H. (2011). Functional relevance of pre-supplementary motor areas for the choice to stop during Stop signal task. Neurosci Res, 70(3), 277–284. doi: 10.1016/j.neures.2011.03.007

Tabu, H., Mima, T., Aso, T., Takahashi, R., & Fukuyama, H. (2012). Common inhibitory prefrontal activation during inhibition of hand and foot responses. Neuroimage, 59(4), 3373–3378. doi: 10.1016/j.neuroimage.2011.10.092

van der Meer, L., Groenewold, N. A., Pijnenborg, M., & Aleman, A. (2013). Psychosis-proneness and neural correlates of self-inhibition in theory of mind. PLoS One, 8(7), e67774. doi: 10.1371/journal.pone.0067774

van Eijk, J., Sebastian, A., Krause-Utz, A., Cackowski, S., Demirakca, T., Biedermann, S. V., … Tuscher, O. (2015). Women with borderline personality disorder do not show altered BOLD responses during response inhibition. Psychiatry Res, 234(3), 378–389. doi: 10.1016/j.pscychresns.2015.09.017

Walther, S., Goya-Maldonado, R., Stippich, C., Weisbrod, M., & Kaiser, S. (2010). A supramodal network for response inhibition. Neuroreport, 21(3), 191–195. doi: 10.1097/WNR.0b013e328335640f

White, T. P., Loth, E., Rubia, K., Krabbendam, L., Whelan, R., Banaschewski, T., … IMAGEN Consortium. (2014). Sex differences in COMT polymorphism effects on prefrontal inhibitory control in adolescence. Neuropsychopharmacology: Official Publication of the American College of Neuropsychopharmacology, 39(11), 2560–2569. doi: 10.1038/npp.2014.107

Wilbertz, T., Deserno, L., Horstmann, A., Neumann, J., Villringer, A., Heinze, H. J., … Schlagenhauf, F. (2014). Response inhibition and its relation to multidimensional impulsivity. Neuroimage, 103, 241–248. doi: 10.1016/j.neuroimage.2014.09.021

Xu, B., Levy, S., Butman, J., Pham, D., Cohen, L. G., & Sandrini, M. (2015). Effect of foreknowledge on neural activity of primary ‘go’ responses relates to response stopping and switching. Front Hum Neurosci, 9, 34. doi: 10.3389/fnhum.2015.00034

Xu, K. Z., Anderson, B. A., Emeric, E. E., Sali, A. W., Stuphorn, V., Yantis, S., & Courtney, S. M. (2017). Neural Basis of Cognitive Control over Movement Inhibition: Human fMRI and

Primate Electrophysiology Evidence. Neuron, 96(6), 1447-1458 e6. doi: 10.1016/j.neuron.2017.11.010

Xue, G., Aron, A. R., & Poldrack, R. A. (2008). Common neural substrates for inhibition of spoken and manual responses. Cereb Cortex, 18(8), 1923–1932. doi: 10.1093/cercor/bhm220

Zandbelt, B. B., van Buuren, M., Kahn, R. S., & Vink, M. (2011). Reduced proactive inhibition in schizophrenia is related to corticostriatal dysfunction and poor working memory. Biol Psychiatry, 70(12), 1151–1158. doi: 10.1016/j.biopsych.2011.07.028

Zandbelt, B. B., & Vink, M. (2010). On the role of the striatum in response inhibition. PLoS One, 5(11), e13848. doi: 10.1371/journal.pone.0013848

Zheng, D., Oka, T., Bokura, H., & Yamaguchi, S. (2008). The key locus of common response inhibition network for no-go and stop signals. J Cogn Neurosci, 20(8), 1434–1442. doi: 10.1162/jocn.2008.20100
